# Swelling-induced calcium signaling in wild-type and *Mlc1*-null astrocytes: paradoxical effects of pharmacological TRPV4 and Piezo1 inhibition

**DOI:** 10.3389/fncel.2026.1908522

**Published:** 2026-08-13

**Authors:** Quinty Bisseling, Vera Kuhnke, Maria S. Brignone, Elena Ambrosini, Huibert D. Mansvelder, Marjo S. van der Knaap, Rogier Min

**Affiliations:** 1 Department of Child Neurology, Amsterdam Leukodystrophy Center, Emma Children’s Hospital, Amsterdam University Medical Center, Amsterdam Neuroscience, Vrije Universiteit Amsterdam, Amsterdam, Netherlands; 2 Department of Integrative Neurophysiology, Center for Neurogenomics and Cognitive Research, Vrije Universiteit Amsterdam, Amsterdam Neuroscience, Amsterdam, Netherlands; 3 Department of Neuroscience, Istituto Superiore di Sanità, Rome, Italy; 4 Department of Neurology, Epileptology, Uniklinik RWTH, Aachen, Germany

**Keywords:** astrocyte, calcium, MLC, MLC1, Piezo1, TRPV4, volume regulation

## Abstract

**Introduction:**

Astrocytes play a crucial role in brain ion and water homeostasis and continuously adapt their volume in response to osmotic challenges. This process is disturbed in the leukodystrophy megalencephalic leukoencephalopathy with subcortical cysts (MLC). Intracellular calcium signaling in response to cell swelling has been implicated in astrocytic volume regulation.

**Methods:**

We examined swelling-induced calcium signals in cultured astrocytes isolated from brains of wild-type and *Mlc1*-null mice, utilizing a microplate reader and Fura-2AM-based imaging.

**Results:**

We showed that wild-type astrocytes respond to hypotonic shocks with a robust calcium signal proportional to the magnitude of the shock. Pharmacological experiments indicated that these responses reflect a combination of intracellular store-mediated calcium release and channel-mediated calcium influx. *Mlc1*-null astrocytes showed an increased baseline calcium level and a slower calcium response to large hypotonic shocks. Unexpectedly, pharmacological inhibition of mechanosensitive cation channels with the TRPV4 antagonist HC-067047 and the mechanosensitive channel inhibitor GsMTx4 did not reduce swelling-induced calcium signals but instead led to a paradoxical increase in amplitude and speed of calcium signals in wild-type and *Mlc1*-null astrocytes, with differences in the magnitude of alteration between the two genotypes.

**Discussion:**

Together, our results suggest subtle alterations in calcium signaling that might affect the volume sensing machinery of *Mlc1*-null astrocytes, conforming with earlier studies that show mistuning of volume-regulated ion channels in MLC. The observed paradoxical effects are consistent with a role for TRPV4- and GsMTx4-sensitive pathways in setting basal responsiveness of astrocytes to osmotic stress, and argue against TRPV4 or Piezo1 being the main direct calcium entry routes during swelling. Overall, our findings highlight the complexity of astrocytic volume regulation in response to hypotonicity.

## 1 Introduction

Astrocytes are the most abundant glial cells in the central nervous system. They are important players in the maintenance of ion and water homeostasis. This is particularly important in the brain, which is encased by a rigid skull and has limited capacity to accommodate volume changes. Even minor brain swelling can be life threatening. Swelling can arise from the accumulation of ions, which drives the movement of osmotically obliged water. During periods of increased neuronal activity, astrocytes take up excess ions and other substances such as neurotransmitters from the extracellular space and redistribute them within the greater panglial syncytium. This process is accompanied by transient astrocyte swelling. Under physiological conditions, swelling is counteracted by activation of the regulatory volume decrease (RVD) process, which restores cell volume (Jentsch, 2016). Activation of the volume-regulated anion channel (VRAC) in response to swelling is an important step in RVD (Formaggio et al., 2019).

While astrocyte volume regulation is tightly controlled, the underlying cellular machinery is not completely understood. Intracellular calcium signaling has been proposed to play a role in this process, although its involvement remains debated (O'Connor and Kimelberg, 1993; Pasantes-Morales et al., 2006; Benfenati et al., 2011). Astrocytes generate complex intracellular calcium signals throughout their cell body and processes, which can drive downstream effects such as blood flow regulation through astrocyte endfeet (for extensive reviews, see Bazargani and Attwell, 2016; Lim et al., 2021; Ahrens et al., 2024). These signals often consist of both calcium influx across the plasma membrane and calcium release from intracellular stores of the endoplasmic reticulum (ER). Release from the ER is mediated through calcium-induced calcium release (CICR), or activation of G protein-coupled receptors (GPCRs). GPCR activation stimulates phospholipase C (PLC), leading to the generation of IP_3_ (1,4,5-trisphosphate), which binds to its receptor on the ER and triggers calcium release (Verkhratsky and Kettenmann, 1996).

Calcium influx through ion channels can be induced by hyposhock-induced cell swelling (O'Connor and Kimelberg, 1993; Fischer et al., 1997). Mechanosensitive cation channels are candidate mediators of such swelling-induced calcium influx. The transient receptor potential vanilloid 4 (TRPV4) channel is activated by osmotic and mechanical stimuli (Liedtke et al., 2000; Strotmann et al., 2000; Wissenbach et al., 2000; Delany et al., 2001; White et al., 2016), and has been shown to mediate calcium influx following hypotonic swelling in primary rat cortical astrocytes (Benfenati et al., 2007). Together with aquaporin-4 (AQP4), TRPV4 has been proposed to form a functional unit involved in astrocyte volume regulation (Benfenati et al., 2011; Jo et al., 2015). The cation channel Piezo1 is intrinsically mechanosensitive (Syeda et al., 2016), and can be activated by mechanical deformation such as membrane stretch or pressure (Coste et al., 2010; Chen et al., 2018). Activation of Piezo1, either pharmacologically or by mechanical stimulation, leads to an increase in intracellular calcium in astrocytes (Velasco-Estevez et al., 2020; Liu et al., 2021; Chi et al., 2022; Gomez-Cruz et al., 2024). Based on these observations, TRPV4 and Piezo1 are plausible contributors to swelling-induced calcium signaling in astrocytes.

Dysfunction of ion and water homeostasis and impaired astrocyte volume regulation are central features of the leukodystrophy megalencephalic leukoencephalopathy with subcortical cysts (MLC, OMIM 604004) (Ridder et al., 2011; van der Knaap et al., 2012). Patients present with macrocephaly and develop chronic white matter edema, cognitive and motor disability, and epilepsy (van der Knaap et al., 1995; Singhal et al., 1996). MLC is caused by variants in *MLC1*, *GLIALCAM*, *AQP4* or *GPRC5B*, which encode membrane proteins localized to astrocyte endfeet (Leegwater et al., 2001; López-Hernández et al., 2011; Passchier et al., 2023). Bi-allelic recessive loss-of-function variants in *MLC1* are the cause of disease in ~75% of MLC patients, and *Mlc1*-null mice recapitulate the major features of the human disease (Boor et al., 2006; Dubey et al., 2015; Passchier et al., 2024). In MLC, fluid accumulates in vacuoles within myelin, and astrocyte endfeet also have a swollen appearance (van der Knaap et al., 2012; Dubey et al., 2015). Astrocytes in MLC models show impaired volume regulation, which has been attributed to dysfunction of VRAC and TRPV4 (Ridder et al., 2011; Lanciotti et al., 2012; Dubey et al., 2015; Jentsch, 2016; Passchier et al., 2023). Previous studies suggest a link between MLC1 and astrocyte calcium signaling. MLC disease models show altered calcium dynamics in response to different types of stimuli (Lanciotti et al., 2012; Petrini et al., 2013; Lanciotti et al., 2016). TRPV4-mediated calcium influx following a hypotonic shock is potentiated by MLC1 overexpression and reduced by mutations that impair its membrane localization (Lanciotti et al., 2012). In addition, MLC1 is regulated by calcium-dependent phosphorylation via CaMKII, the calcium/calmodulin-dependent protein kinase 2, which has been linked to modulation of VRAC and RVD (Brignone et al., 2022; Brignone et al., 2024). Together, these findings point to a connection between calcium signaling and impaired volume regulation in MLC.

In this study, we examined swelling-induced calcium dynamics in cultured primary astrocytes from wild-type and *Mlc1*-null mice, an established mouse model of MLC (Dubey et al., 2015). Using high-throughput ratiometric calcium imaging with Fura-2AM in a microplate reader, we characterized the temporal properties and sources of calcium signals following hypotonic stimulation. We assessed the relative contributions of extracellular calcium influx and intracellular stores and examined the involvement of the mechanosensitive channels TRPV4 and Piezo1 using pharmacological approaches. Together, this study allowed us to define how MLC1 and mechanosensitive ion channels modulate the responsiveness of astrocytes to osmotic stress.

## 2 Materials and methods

### 2.1 Animal and cell models

Experiments were performed on HEK293 cells and cultured primary astrocytes. Primary astrocytes were obtained from wild-type mice and transgenic *Mlc1*-null mice. All mice had a C57Bl/6J background. Generation of *Mlc1*-null mice is described in (Dubey et al., 2015). Wild-type and *Mlc1*-null mice were obtained by homozygous breeding of *Mlc1*^wt/wt^ x *Mlc1*^wt/wt^ or *Mlc1*^null/null^ x *Mlc1*^null/null^ mice, respectively. Breeding pairs were regularly refreshed from heterozygous breeding to limit drift in genetic background. Experimental procedures involving mice were in strict compliance with animal welfare policies of the Dutch government and were approved by the Institutional Animal Care and Use Committee of the Amsterdam University Medical Center, location AMC, Amsterdam.

### 2.2 Cell culture of HEK293 cells

HEK293 cells were cultured in Dulbecco’s Modified Eagle Medium/Nutrient Mixture F-12 (1:1) (DMEM/F-12 (1:1)) with GlutaMAX™-I and Phenol Red (Gibco, 31331-028), supplemented with 10% Fetal Bovine Serum (FBS; Gibco, 10270-106) and 1% Penicillin–Streptomycin (Pen Strep; Gibco, 15140122) in a humidified incubator (37 °C / 5% CO_2_). At a confluency of >80%, cells were seeded on poly-L-lysine (PLL)-coated (0.1 mg/mL for 1–2 h at 37 °C; Sigma, P2636) 96-well plates (black, clear bottom; Greiner, 655090) with a cell density of 20.000 cells per well. Plates were used for experiments between 24 and 72 h after plating.

### 2.3 Isolation and cell culture of primary astrocytes

Cortical primary astrocytes were isolated from neonatal wild-type and *Mlc1*-null mice on postnatal day 6 to day 9 (p6-p9) (protocol adapted from (McCarthy and De Vellis, 1980)). Brains were isolated from the skull and cerebral cortices were dissected by removing the olfactory bulb, cerebellum, midbrain, and meninges. This was done in ice-cold, sterile Hanks’ Balanced Salt Solution without calcium and magnesium, with Phenol Red (HBSS^−/−^; Gibco, 14,170–088) supplemented with 1% Pen Strep. The obtained neocortical tissue was minced with a sterile scalpel and incubated in TrypLE™ Express (1X; Gibco, 12605-010) supplemented with DNase I (40 μg/mL; Roche, 11284932001) in a rotator device for 25 min at 37 °C. The cell suspension was centrifuged (475 x *g*, 3 min) at room temperature (RT) and the supernatant was removed. The pellet was resuspended in complete primary astrocyte medium (DMEM/F-12 (1:1) with GlutaMAX™-I and Phenol Red (Gibco, 31331-028), 10% FBS, 1% Sodium Pyruvate (100 mM; Gibco, 11360-039) and 1% Pen Strep) and centrifuged again (475 x *g*, 3 min, RT). For mechanical dissociation, the cell pellet was titrated in complete primary astrocyte medium using a 5 mL and 2 mL pipette, respectively. In between and after the titration steps, the cell suspension was centrifuged (475 x *g*, 3 min, RT) and resuspended in complete primary astrocyte medium. The cell suspension was filtered through a 70 μm Nylon Cell Strainer (Corning, 431751) and centrifuged (475 x *g*, 5 min, RT). Finally, the cell pellet was resuspended in complete primary astrocyte medium and transferred into PLL-coated (0.1 mg/mL for 1–2 h at 37 °C) T75 flasks and incubated in a humidified incubator (37 °C / 5% CO_2_). Until >80% confluency was reached (~1 week), cells were washed with Dulbecco’s Phosphate Buffered Saline (DPBS; Gibco, 14190-094) and medium was changed every 2–3 days. At >80% confluency, contaminating oligodendrocytes, microglia and precursor cells were dislodged overnight (ON) by orbital shaking (180 RPM; VWR, 89032-088). The next day, flasks were vigorously shaken by hand for 30 s and washed in DPBS three times. Complete primary astrocyte medium was added, and cells were used in experiments or maintained for a week maximum in a humidified incubator (37 °C / 5% CO_2_) with medium changes every 3 days.

### 2.4 Reagents

Isotonic solution containing (in mM) 140 NaCl, 4 KCl, 2 MgCl_2_, 2 CaCl_2_, 10 HEPES, 5 D(+)-Glucose (pH adjusted to 7.35 with NaOH and osmolality adjusted to 317 mOsm/kg) was used for all calcium imaging experiments. Zero calcium solution containing (in mM) 140 NaCl, 4 KCl, 2 MgCl_2_, 10 HEPES, 5 D(+)-Glucose, 2.5 EGTA (ethylene glycol-bis(*β*-aminoethyl ether)-N,N,N′,N′-tetraacetic acid) (pH adjusted to 7.35 with NaOH and osmolality adjusted to 316 mOsm/kg) was used for zero calcium conditions. Ionomycin (Abcam, ab120116; 3.3 mM stock prepared in dimethyl sulfoxide (DMSO; (PanReac AppliChem, A3672.0100)) was diluted 1:10 in HBSS with calcium and magnesium, without Phenol Red (HBSS^+/+^; Gibco, 14025-092) to reach the final concentration of 0.33 mM. The SERCA inhibitor thapsigargin (Tebu-Bio, 10-2105; 2 mM stock prepared in DMSO) was diluted in isotonic solution to a 2 and 5 μM working solution. The PLC inhibitor U-73122 (1-[6-[[(17β)-3-Methoxyestra-1,3,5(10)-trien-17-yl]amino]hexyl]-1H-pyrrole-2,5-dione; Bio-Connect, 112648–68-7; 10 mM stock prepared in DMSO) was diluted in isotonic solution to a 5 μM working solution. TRPV4 antagonist HC-067047 (2-methyl-1-[3-(4-morpholinyl)propyl]-5-phenyl-N-[3-(trifluoromethyl)-phenyl]-1H-pyrrole-3-carboxamide; Tebu-Bio, T4680; 4 mM stock prepared in DMSO) (Everaerts et al., 2010), and GsMTx4 (Grammostola spatulata mechanotoxin 4, M-theraphotoxin-Gr1a, M-TRTX-Gr1a; MedChemExpress, HY-P1410; 4 mM stock prepared in DMSO), which is the inhibitor of cation-permeable mechanosensitive channels Piezo1 and TRP channels (Bae et al., 2011), were diluted to 4 μM in isotonic solution before use.

### 2.5 Western blots

Cell pellet preparation and western blotting procedures were performed as described previously (Bisseling et al., 2026). The following antibodies were used: rabbit anti-TRPV4 (1:500; Santa Cruz Biotechnology, USA), and mouse anti-GAPDH (1:1000; Santa Cruz Biotechnology, USA).

### 2.6 Calcium imaging with Fura-2AM

Calcium imaging was conducted with the ratiometric calcium indicator dye Fura-2AM (5 mM stock prepared in DMSO; ThermoScientific, F1221) (Grynkiewicz et al., 1985) using a CLARIOstar*^Plus^* Microplate Reader (type 0430; BMG LABTECH, Germany). HEK293 cells or primary astrocytes were washed with DPBS and incubated with 10 μM Fura-2AM diluted in HEK293 medium or complete primary astrocyte medium for 30 min at 37 °C. Afterwards cells were washed with DPBS and kept in 100 μL isotonic solution for imaging. Imaging methods were optimized using HEK293 cells. As a positive control of the dye, HEK293 cells loaded with Fura-2AM showed a robust intracellular calcium increase upon application of ionomycin (0.33 mM; Supplementary Figure 1), a calcium ionophore that stimulates calcium release from intracellular stores (Liu and Hermann, 1978; Morgan and Jacob, 1994). The total imaging time per well was kept at 300 s, and fluorescence measurements were done through bottom reading at excitation wavelengths 340 and 380 nm every 1.63 s. This imaging speed allowed us to distinguish relevant changes in calcium signal and observe the hypotonic shock-induced calcium peak. Hypotonic shocks were administered to the cells after a 30 s baseline recording period. Hypotonic shocks were induced by injecting water (MilliQ; 43 μL for a 30% hypotonic shock, 67 μL for 40%, 100 μL for 50%) with a low injection speed of 65 μL/s to prevent cell detachment from the bottom of the well. A shaking step (double orbital, 100 RPM, 1 s) was included after water injection to ensure proper mixing of the injected fluid, thereby providing precise and fast hypotonic shock conditions. Fura-2AM F340/F380 ratios were calculated from raw fluorescence signal data for the respective wavelengths. Experiments were performed sequentially for all loaded wells in a plate (1–24 wells per plate), which was kept at RT. Wells were scanned with spiral averaging, with a diameter of 3 mm. For all experimental manipulations, control wells were interspersed in the same plate. For experiments with the zero extracellular calcium condition, astrocytes were washed with zero calcium solution and kept in fresh zero calcium solution during the imaging period. For experiments with thapsigargin, wells were incubated with 2 or 5 μM thapsigargin for 30 min at RT. Following incubation, a maximum of 4 wells with thapsigargin were sequentially measured to limit the exposure of the cells to thapsigargin to maximally 1 h. U-73122 (5 μM, 5 min, RT), HC-067047 (4 μM, 4 min, RT) and GsMTx4 (4 μM, 4 min, RT), were added to the wells before imaging and kept on the wells during the whole imaging period to ensure optimal binding. For all drugs, equal concentrations were added to the MilliQ used for injections to prevent drug dilution upon hypotonic shock application. To account for potential osmolarity changes or non-specific effects caused by DMSO, an equal amount of DMSO (1:1000) was added to control wells which were measured interspersed with drug-treated wells.

### 2.7 Data analysis and statistics

**Figure 1:**
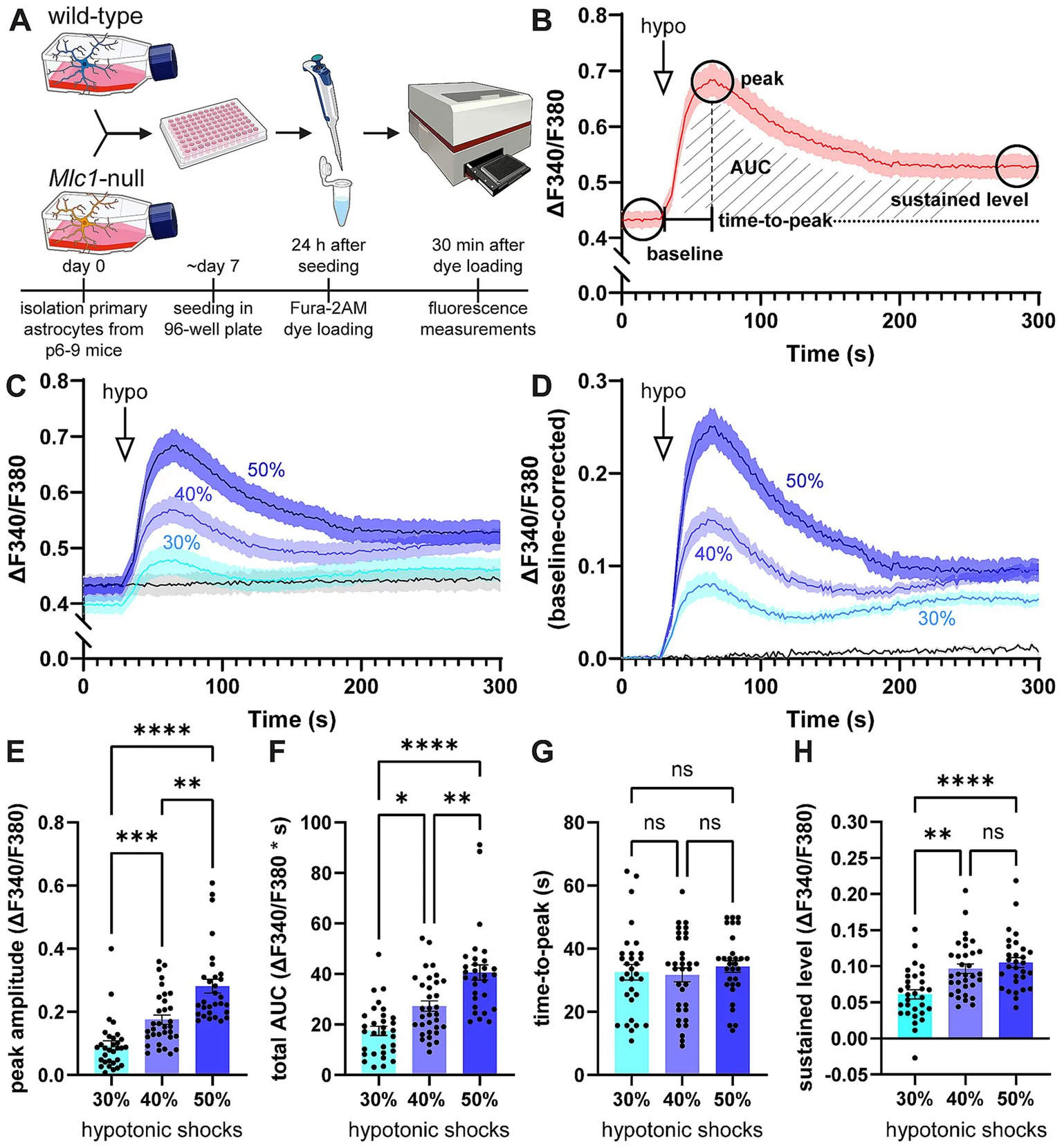
Calcium responses to increasing hypotonic shocks in primary wild-type astrocytes. **(A)** Schematic workflow of calcium imaging experiments on cultured primary mouse astrocytes. Illustration from NIAID NIH BioArt Source (bioart.niaid.nih.gov/bioart/##). ## corresponds with used images (7, 40, 143, 161, 303, and 408). **(B)** Parameters extracted from intracellular calcium traces are highlighted: Baseline, area under the curve (AUC), time-to-peak (TTP), and sustained calcium level, with corresponding bar graphs in **(E–H)**, respectively. **(C, D)** Averaged intracellular calcium traces in wild-type astrocytes exposed to a 30% (cyan), 40% (blue), or 50% (dark blue) hypotonic shock applied after 30 s (black arrow). Traces in **(D)** are baseline-corrected. **(E)** The peak amplitude of the calcium signal in response to increasing hypotonic shock conditions (30%: 0.09399 ± 0.01402 ΔF340/F380, 40%: 0.1758 ± 0.01468 ΔF340/F380, 50%: 0.2817 ± 0.02205 ΔF340/F380; 30% vs. 40%, adj. *p* = 0.0009, 30% vs. 50%, adj. *p* < 0.0001, 40% vs. 50%, adj. *p* = 0.0028). **(F)** The total AUC of the calcium signal in response to increasing hypotonic shock conditions (30%: 17.50 ± 1.834 ΔF340/F380 * s, 40%: 27.29 ± 2.047 ΔF340/F380 * s, 50%: 40.65 ± 2.994 ΔF340/F380 * s; 30% vs. 40%, adj. *p* = 0.012, 30% vs. 50%, adj. *p* < 0.0001, 40% vs. 50%, adj. *p* = 0.0028). **(G)** The TTP of the calcium signal in response to increasing hypotonic shock conditions (30%: 32.58 ± 2.455 s, 40%: 31.68 ± 2.215 s, 50%: 34.38 ± 1.879 s; 30% vs. 40%, adj. *p* > 0.9999, 30% vs. 50%, adj. *p* > 0.9999, 40% vs. 50%, adj. *p* > 0.9999). **(H)** The sustained calcium level, based on the last 10 measurements of the traces (285.3 - 300 s), after exposure to hypotonic shock conditions (30%: 0.06099 ± 0.006361 ΔF340/F380, 40%: 0.09645 ± 0.006494 ΔF340/F380, 50%: 0.1050 ± 0.006962 ΔF340/F380; 30% vs. 40%, adj. *p* = 0.0015, 30% vs. 50%, adj. *p* < 0.0001, 40% vs. 50%, adj. *p* > 0.9999). All data is presented as mean ± SEM. Statistical analyses in **(E–H)** were done using a Kruskal-Wallis test with Dunn’s multiple comparison correction. Dots in bar graphs indicate individual values of wells (30%: *n* = 31 wells, 40%: *n* = 33 wells, 50%: *n* = 30 wells). Individual intracellular traces are shown in Supplementary Figure 3.

**Figure 2:**
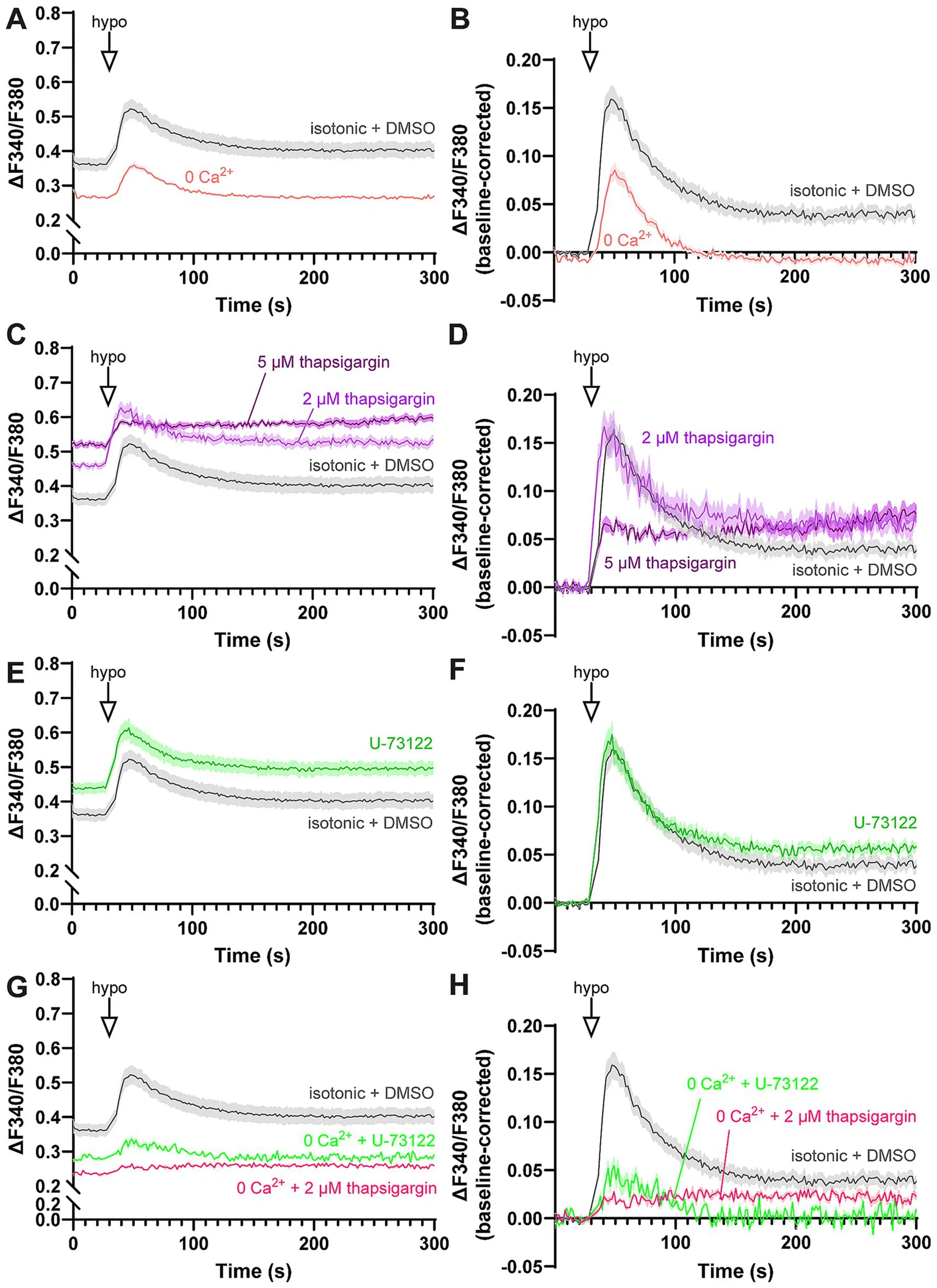
The contribution of extracellular calcium and calcium release from intracellular stores to swelling-induced calcium signals in primary wild-type astrocytes. **(A–H)** Averaged intracellular calcium traces in wild-type astrocytes exposed to a 50% hypotonic shock (black arrow). Traces in **(B, D, F, H)** are baseline-corrected. Wild-type astrocytes were pretreated and during imaging kept in zero calcium solution **(A, B)**, SERCA inhibitor thapsigargin **(C, D)**, phospholipase C inhibitor U-73122 **(E, F)** or a combination of thapsigargin or U-73122 with the zero calcium solution **(G**, **H)**, with isotonic solution with DMSO as a control. All data is presented as mean ± SEM. Individual intracellular traces are shown in Supplementary Figure 4.

CLARIOstar*^Plus^* data was recorded using SMART Control software. Data was processed using Microsoft Excel. Raw fluorescence intensity measurements using an excitation wavelength of 340 nm (F340) and 380 nm (F380) were extracted. Fluorescence values from unloaded wells (not incubated with Fura-2AM) were subtracted from every measurement to account for baseline (auto)fluorescence and background signal not related to the calcium dye. With these corrected values the F340/380 ratio was calculated for each time point. Data representation and statistical analyses were done using GraphPad Prism version 10.2.0 for Windows (GraphPad Software, Boston, Massachusetts USA). Properties of baseline and swelling-induced calcium dynamics were defined as follows: Peak amplitude was defined as the distance between the baseline and the highest F340/F380 ratio observed within 100 s after measurement start. Total area under the curve (AUC) was defined as the total area between the curve and the extended baseline. Time-to-peak (TTP) was defined as the time between the moment of water injection (30 s) and the moment of peak amplitude. Sustained calcium levels were defined as the mean F340/F380 ratio during the last 10 measurement points (285.3 s–300 s), from which the baseline is subtracted. Baseline calcium levels were defined as the mean F340/F380 ratio during the first 30 s of a recording, or the first 18 measurement points, before water injection. For determination of baseline calcium levels, only measurements from the first 5–6 wells per plate per condition were included. For baseline comparisons, data obtained from all hypotonic shock data sets (where applicable 30% and/or 40% and/or 50%) was included. For all other parameters (besides Figures 1, 2), only data from 50% hypotonic datasets was used. Baseline calcium levels were compared using either Student’s *t*-test, Mann–Whitney test, one-way ANOVA with multiple comparisons or Kruskal-Wallis test with Dunn’s correction, depending on whether data passed normality testing. To compare peak amplitudes, total AUC, TTP, and sustained (calcium) levels amongst different hypotonic shocks and genotypes, one-way ANOVAs with multiple comparison correction (Kruskal-Wallis test with Dunn’s correction) were carried out. Statistical analysis of western blot data was done with a Student’s *t*-test. Statistically significant differences were defined as * (≤0.05), ** (≤0.01), *** (≤0.001), **** (≤0.0001). Data points in graphs represent the measurement from a single well. Four to six plate runs were done for each condition per genotype. Data are expressed as mean ± SEM.

**Figure 5:**
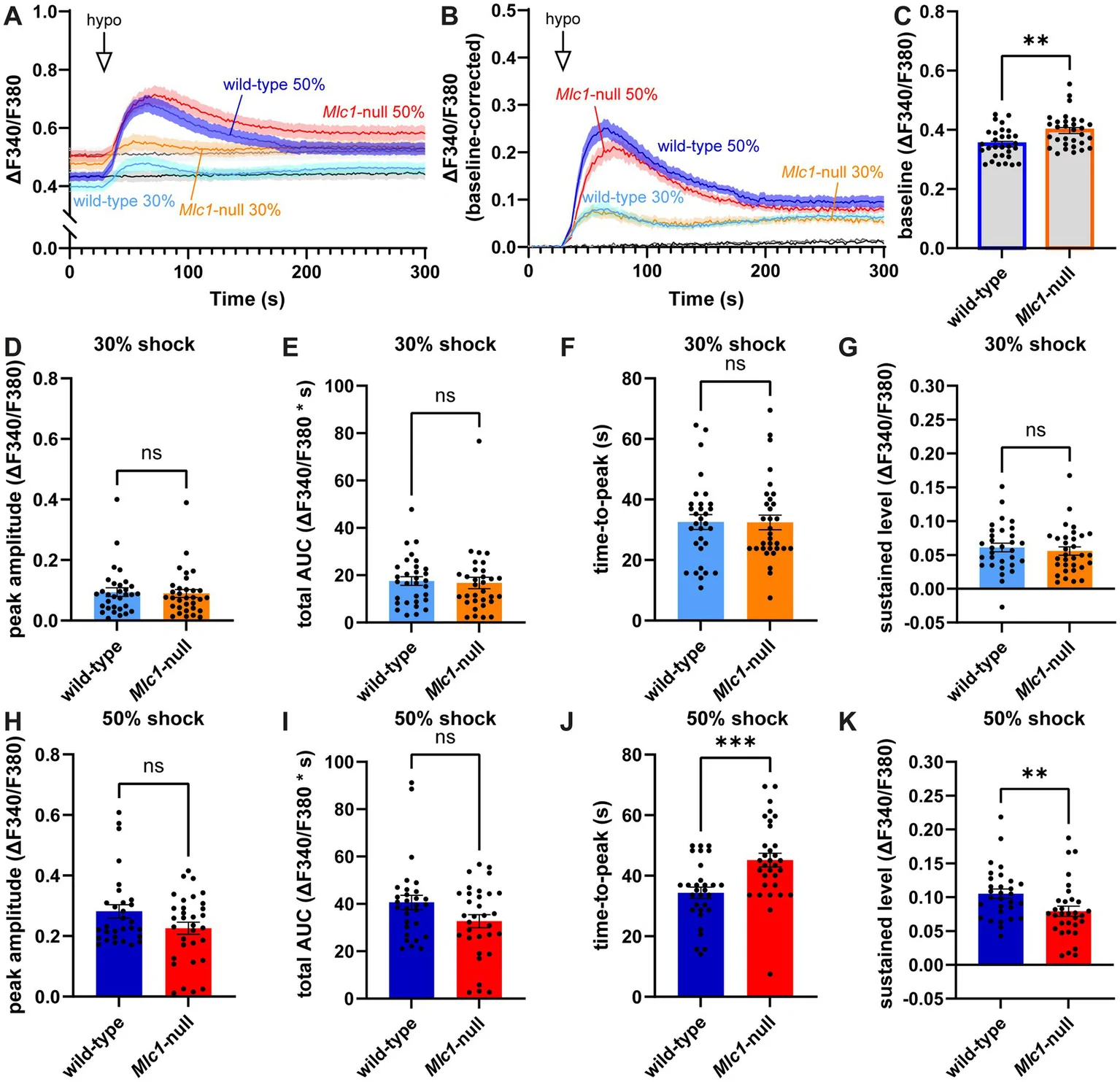
Altered baseline and swelling-induced calcium signals in *Mlc1*-null astrocytes. **(A, B)** Averaged intracellular calcium traces in wild-type (cyan, blue) and *Mlc1*-null (orange, red) astrocytes exposed to a 30% (cyan, orange) or 50% (blue, red) hypotonic shock (black arrow). Traces in **(B)** are baseline-corrected. **(C)** Baseline calcium levels of wild-type and *Mlc1*-null astrocytes (wild-type: 0.3519 ± 0.009295 ΔF340/F380, *n* = 30 wells, *Mlc1*-null: 0.3977 ± 0.01022 ΔF340/F380, *n* = 31 wells, *p* = 0.0039). **(D–K)** Different parameters of the calcium response to a 30% **(D–G)** or 50% **(H–K)** hypotonic shock in wild-type and *Mlc1*-null astrocytes (wild-type 30%: *n* = 31 wells, *Mlc1*-null 30%: *n* = 32 wells; wild-type 50%: *n* = 30 wells, *Mlc1*-null 50%: *n* = 32 wells). **(D, H)** The peak amplitude (wild-type 30%: 0.09399 ± 0.01402 ΔF340/F380, *Mlc1*-null 30%: 0.08936 ± 0.01321 ΔF340/F380, *p* = 0.7380; wild-type 50%: 0.2817 ± 0.02205 ΔF340/F380, *Mlc1*-null 50%: 0.2258 ± 0.01990 ΔF340/F380, *p* = 0.3371). **(E, I)** The total AUC (wild-type 30%: 17.50 ± 1.834 ΔF340/F380 * s, *Mlc1*-null 30%: 16.72 ± 2.412 ΔF340/F380 * s, *p* = 0.5342; wild-type 50%: 40.65 ± 2.994 ΔF340/F380 * s, *Mlc1*-null 50%: 32.71 ± 2.719 ΔF340/F380 * s, *p* = 0.2077). **(F, J)** The time-to-peak (TTP) (wild-type 30%: 32.58 ± 2.455 s, *Mlc1*-null 30%: 32.44 ± 2.418 s, *p* = 0.7870; wild-type 50%: 34.38 ± 1.879 s, *Mlc1*-null 50%: 45.18 ± 2.286 s, *p* = 0.0008). **(G, K)** The sustained calcium level (wild-type 30%: 0.06099 ± 0.006361 ΔF340/F380, *Mlc1*-null 30%: 0.05577 ± 0.006039 ΔF340/F380, *p* = 0.4399; wild-type 50%: 0.1050 ± 0.006962 ΔF340/F380, *Mlc1*-null 50%: 0.07920 ± 0.007377 ΔF340/F380, *p* = 0.0061). All data is presented as mean ± SEM. Statistical analyses in **(C–K)** were done using a Mann–Whitney test. Dots in bar graphs indicate individual values of wells. Individual intracellular traces of *Mlc1*-null astrocytes are shown in Supplementary Figure 6.

## 3 Results

### 3.1 Hypotonic shocks induce intracellular calcium responses in primary astrocytes

Astrocytes respond to hypotonicity-induced cell swelling with an increase in intracellular calcium concentration (O'Connor and Kimelberg, 1993; Benfenati et al., 2011). We investigated astrocyte calcium signals in response to different hypotonic shocks, ranging from 30 to 50%. Cultured primary murine astrocytes were loaded with the ratiometric calcium indicator Fura-2AM and intracellular calcium was measured using a plate reader (see Materials and Methods; for workflow see Figure 1A). Baseline calcium levels were determined for 30 s, after which hypotonic shocks were administered with water injections. Multiple parameters were extracted from the resulting dynamic calcium measurements (Figure 1B): The average baseline intracellular calcium level, the peak of the response following the hypotonic shock, the AUC indicating the integrated size of the entire calcium response, the TTP and the sustained level reflecting slow calcium increases.

Astrocytes responded to a hypotonic shock with a transient increase in intracellular calcium (Figure 1C; baseline-corrected curves in Figure 1D). The size of the calcium response scaled with the magnitude of the hypotonic shock. The averaged calcium response often showed an initial increase followed by a slower sustained calcium increase. This was particularly visible using 30–40% hypotonic shocks, with the phases overlapping for 50% hypotonic shocks. Peak amplitude and total AUC increased proportional to the size of the hypotonic shock (30, 40% or 50%; Figures 1E,F). The TTP was not significantly different between hypotonic conditions (Figure 1G), suggesting that the timing of the calcium response is relatively insensitive to stimulus strength. The sustained calcium level was increased for the 40% shock when compared to 30% but did not show a further increase for 50% (Figure 1H). In conclusion, primary astrocytes respond to hypotonic shocks with a calcium response proportional to the shock strength, while the timing of the response remains largely unchanged.

### 3.2 Hypotonic shock-induced calcium signals in primary astrocytes are mediated by a combination of intracellular stores and ion channels

**Figure 3:**
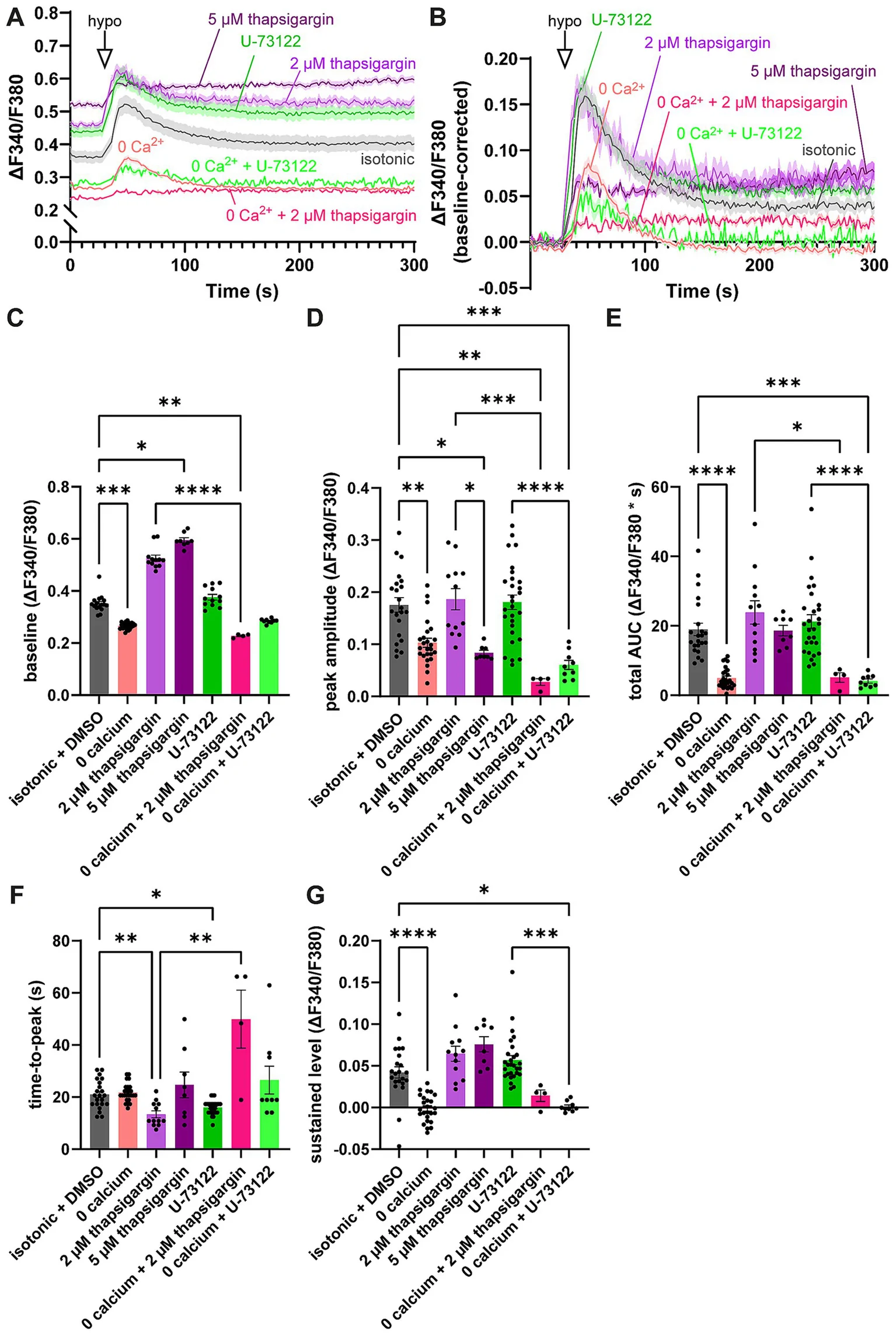
Extracellular calcium and calcium release from intracellular stores contribute to swelling-induced calcium signals in primary wild-type astrocytes. **(A, B)** Combined averaged intracellular calcium traces (same traces as in Figure 2) in wild-type astrocytes exposed to a 50% hypotonic shock (black arrow). Traces in **(B)** are baseline-corrected. **(C)** Baseline calcium levels of wild-type astrocytes treated with zero calcium solution, thapsigargin (2 μM or 5 μM), U-73122 or a combination of conditions (isotonic + DMSO: 0.3516 ± 0.008381 ΔF340/F380, *n* = 16 wells, 0 calcium: 0.2661 ± 0.002376 ΔF340/F380, *n* = 25 wells, 2 μM thapsigargin: 0.5257 ± 0.01193 ΔF340/F380, *n* = 12 wells, 5 μM thapsigargin: 0.5947 ± 0.009696 ΔF340/F380, *n* = 8 wells, U-73122: 0.3761 ± 0.01082 ΔF340/F380, *n* = 12 wells, 0 calcium + 2 μM thapsigargin: 0.2279 ± 0.003343 ΔF340/F380, *n* = 4 wells, 0 calcium + U-73122: 0.2846 ± 0.00285 ΔF340/F380, *n* = 9 wells; isotonic + DMSO vs. 0 calcium, adj. *p* = 0.0006, isotonic + DMSO vs. 2 μM thapsigargin, adj. *p* = 0.1594, isotonic + DMSO vs. 5 μM thapsigargin, adj. *p* = 0.0481, isotonic + DMSO vs. U-73122, adj. *p* > 0.9999, isotonic + DMSO vs. 0 calcium + 2 μM thapsigargin, adj. *p* = 0.0070, isotonic + DMSO vs. 0 calcium + U-73122, adj. *p* = 0.8065, 0 calcium vs. 0 calcium + 2 μM thapsigargin, adj. *p* > 0.9999, 0 calcium vs. 0 calcium + U-73122, adj. *p* > 0.9999, 2 μM thapsigargin vs. 5 μM thapsigargin, adj. *p* > 0.9999, 2 μM thapsigargin vs. 0 calcium + 2 μM thapsigargin, adj. *p* < 0.0001, U-73122 vs. 0 calcium + U-73122, adj. *p* = 0.32). **(D–G)** Different parameters of the calcium response to a 50% hypotonic shock in wild-type astrocytes treated with zero calcium solution, thapsigargin (2 μM or 5 μM), U-73122 or a combination of conditions (isotonic + DMSO: *n* = 22 wells, 0 calcium: *n* = 25 wells, 2 μM thapsigargin: *n* = 12 wells, 5 μM thapsigargin: *n* = 8 wells, U-73122: *n* = 28 wells, 0 calcium + 2 μM thapsigargin: *n* = 4 wells, 0 calcium + U-73122: *n* = 9 wells). **(D)** The peak amplitude (isotonic + DMSO: 0.1755 ± 0.01389 ΔF340/F380, 0 calcium: 0.1028 ± 0.008685 ΔF340/F380, 2 μM thapsigargin: 0.1865 ± 0.02039 ΔF340/F380, 5 μM thapsigargin: 0.08373 ± 0.004819 ΔF340/F380, U-73122: 0.1807 ± 0.01395 ΔF340/F380, 0 calcium + 2 μM thapsigargin: 0.02746 ± 0.006238 ΔF340/F380, 0 calcium + U-73122: 0.06038 ± 0.008820 ΔF340/F380; isotonic + DMSO vs. 0 calcium, adj. *p* = 0.0093, isotonic + DMSO vs. 2 μM thapsigargin, adj. *p* > 0.9999, isotonic + DMSO vs. 5 μM thapsigargin, adj. *p* = 0.0151, isotonic + DMSO vs. U-73122, adj. *p* > 0.9999, isotonic + DMSO vs. 0 calcium + 2 μM thapsigargin, adj. *p* = 0.0011, isotonic + DMSO vs. 0 calcium + U-73122, adj. *p* = 0.0002, 0 calcium vs. 0 calcium + 2 μM thap sigargin, adj. *p* = 0.3778, 0 calcium vs. 0 calcium + U-73122, adj. *p* = 0.6349, 2 μM thapsigargin vs. 5 μM thapsigargin, adj. *p* = 0.0130, 2 μM thapsigargin vs. 0 calcium + 2 μM thapsigargin, adj. *p* = 0.0009, U-73122 vs. 0 calcium + U-73122, adj. *p* < 0.0001). **(E)** The total AUC (isotonic + DMSO: 18.96 ± 1.792 ΔF340/F380 * s, 0 calcium: 4.979 ± 0.6062 ΔF340/F380 * s, 2 μM thapsigargin: 23.87 ± 3.345 ΔF340/F380 * s, 5 μM thapsigargin: 18.64 ± 1.529 ΔF340/F380 * s, U-73122: 21.27 ± 1.93 ΔF340/F380 * s, 0 calcium + 2 μM thapsigargin: 5.165 ± 1.374 ΔF340/F380 * s, 0 calcium + U-73122: 4.133 ± 0.5834 ΔF340/F380 * s; isotonic + DMSO vs. 0 calcium, adj. *p* < 0.0001, isotonic + DMSO vs. 2 μM thapsigargin, adj. *p* > 0.9999, isotonic + DMSO vs. 5 μM thapsigargin, adj. *p* > 0.9999, isotonic + DMSO vs. U-73122, adj. *p* > 0.9999, isotonic + DMSO vs. 0 calcium + 2 μM thapsigargin, adj. *p* = 0.0617, isotonic + DMSO vs. 0 calcium + U-73122, adj. *p* = 0.0003, 0 calcium vs. 0 calcium + 2 μM thapsigargin, adj. *p* > 0.9999, 0 calcium vs. 0 calcium + U-73122, adj. *p* > 0.9999, 2 μM thapsigargin vs. 5 μM thapsigargin, adj. *p* > 0.9999, 2 μM thapsigargin vs. 0 calcium + 2 μM thapsigargin, adj. *p* = 0.0202, U-73122 vs. 0 calcium + U-73122, adj. *p* < 0.0001). **(F)** The time-to-peak (TTP) (isotonic + DMSO: 20.95 ± 1.167 s, 0 calcium: 21.68 ± 0.6952 s, 2 μM thapsigargin: 13.38 ± 1.35 s, 5 μM thapsigargin: 24.64 ± 4.937 s, U-73122: 15.96 ± 0.4914 s, 0 calcium + 2 μM thapsigargin: 49.90 ± 11.16 s, 0 calcium + U-73122: 26.51 ± 5.339 s; isotonic + DMSO vs. 0 calcium, adj. *p* > 0.9999, isotonic + DMSO vs. 2 μM thapsigargin, adj. *p* = 0.0052, isotonic + DMSO vs. 5 μM thapsigargin, adj. *p* > 0.9999, isotonic + DMSO vs. U-73122, adj. *p* = 0.0114, isotonic + DMSO vs. 0 calcium + 2 μM thapsigargin, adj. *p* = 0.7541, isotonic + DMSO vs. 0 calcium + U-73122, adj. *p* > 0.9999, 0 calcium vs. 0 calcium + 2 μM thapsigargin, adj. *p* > 0.9999, 0 calcium vs. 0 calcium + U-73122, adj. *p* > 0.9999, 2 μM thapsigargin vs. 5 μM thapsigargin, adj. *p* = 0.0851, 2 μM thapsigargin vs. 0 calcium + 2 μM thapsigargin, adj. *p* = 0.0011, U-73122 vs. 0 calcium + U-73122, adj. *p* = 0.1304). **(G)** The sustained calcium level (isotonic + DMSO: 0.04189 ± 0.006880 ΔF340/F380, 0 calcium: −0.001272 ± 0.003231 ΔF340/F380, 2 μM thapsigargin: 0.06443 ± 0.009103 ΔF340/F380, 5 μM thapsigargin: 0.07577 ± 0.009029 ΔF340/F380, U-73122: 0.05683 ± 0.005425 ΔF340/F380, 0 calcium + 2 μM thapsigargin: 0.01406 ± 0.006781 ΔF340/F380, 0 calcium + U-73122: 0.0008566 ± 0.002063 ΔF340/F380; isotonic + DMSO vs. 0 calcium, adj. *p* < 0.0001, isotonic + DMSO vs. 2 μM thapsigargin, adj. *p* > 0.9999, isotonic + DMSO vs. 5 μM thapsigargin, adj. *p* = 0.4262, isotonic + DMSO vs. U-73122, adj. *p* > 0.9999, isotonic + DMSO vs. 0 calcium + 2 μM thapsigargin, adj. *p* = 0.9119, isotonic + DMSO vs. 0 calcium + U-73122, adj. *p* = 0.0146, 0 calcium vs. 0 calcium + 2 μM thapsigargin, adj. *p* > 0.9999, 0 calcium vs. 0 calcium + U-73122, adj. *p* > 0.9999, 2 μM thapsigargin vs. 5 μM thapsigargin, adj. *p* > 0.9999, 2 μM thapsigargin vs. 0 calcium + 2 μM thapsigargin, adj. *p* = 0.1144, U-73122 vs. 0 calcium + U-73122, adj. *p* = 0.0002). All data is presented as mean ± SEM. Statistical analyses in **(C–G)** were done using a Kruskal-Wallis test with Dunn’s multiple comparison correction. Dots in bar graphs indicate individual values of wells, and only statistically significant comparisons are indicated in bar graphs. Individual intracellular traces are shown in Supplementary Figure 4.

We aimed to identify the origin of the hypotonicity-induced calcium responses. We performed experiments in the absence of extracellular calcium to investigate the contribution of cation channel-mediated calcium influx (Figures 2A,B). Maintaining cells in zero calcium extracellular solution decreased baseline intracellular calcium levels (Figure 3C). Following a hypotonic shock, peak amplitude (Figure 3D) and total AUC (Figure 3E) also decreased in zero calcium conditions. The TTP was not affected (Figure 3F), and the sustained calcium level was completely abolished (Figure 3G). This suggests that influx of extracellular calcium, presumably through swelling-induced opening of cation channels, contributes to the swelling-induced calcium response, in particular to the sustained phase.

To investigate the contribution of calcium released from intracellular stores, we treated astrocytes with 2 or 5 μM thapsigargin (Figures 2C,D). Thapsigargin is an inhibitor of the ER calcium ATPase (SERCA), and its application leads to depletion of intracellular ER calcium stores (Treiman et al., 1998). Treatment with 5 μM thapsigargin significantly increased baseline calcium levels (Figure 3C). While treatment with 2 μM thapsigargin resulted in a faster calcium response (Figure 3F), no statistically significant differences were detected in other parameters compared to control. In the 5 μM thapsigargin condition, peak amplitude was significantly decreased to 50% of the control (Figure 3D), while total AUC (Figure 3E), TTP (Figure 3F) and the sustained calcium level (Figure 3G) were not changed.

Release of calcium from intracellular stores, which is often downstream of activation of membrane GPCRs, generally involves PLC signaling (McCudden et al., 2005). U-73122 is a PLC and A2 inhibitor that interferes with IP_3_ receptor-mediated calcium release from the ER (Smith et al., 1990). Treatment with 5 μM U-73122 (Figures 2E,F), did not measurably affect baseline calcium levels (Figure 3C), or peak amplitude, total AUC or the sustained calcium level when compared to the DMSO control (Figure 3D,E,G). Only a slight increase in speed of the response was seen (Figure 3F), however these results suggest that PLC-dependent signaling is not a major contributor under these conditions.

Thapsigargin-induced depletion of ER calcium stores can activate store-operated calcium entry (SOCE) when extracellular calcium is present (Verkhratsky and Parpura, 2014). To better distinguish intracellular store release from extracellular calcium influx, we combined removal of extracellular calcium with either ER store depletion using thapsigargin or inhibition of PLC-dependent calcium release using U-73122 (Figures 2G,H). Zero calcium conditions in combination with 2 μM thapsigargin decreased the baseline calcium levels (Figure 3C), while the AUC (Figure 3E) and the sustained calcium level (Figure 3G) was lower but not significant. The calcium peak, which was still present in zero calcium and 2 μM thapsigargin conditions individually, was almost completely abolished when both conditions were combined (Figure 3D). As there is not a defined peak, the TTP was very variable (Figure 3F). The decrease in total AUC and sustained levels are likely due to the effect of zero calcium instead of thapsigargin, while the peak is affected by both. Zero calcium in combination with U-73122 did not affect baseline levels (Figure 3C) and TTP (Figure 3F), but did decrease peak amplitude (Figure 3D), total AUC (Figure 3E), and the sustained calcium level (Figure 3G). These results are likely because of the zero calcium solution and not U-73122. Figures 3A,B show an overview of (baseline-corrected) intracellular calcium responses for all conditions combined.

Together, these results indicate that both extracellular calcium influx and release from intracellular stores contribute to hypotonicity-induced calcium responses in primary astrocytes. While the initial calcium peak appears to depend on both sources, the sustained calcium response mostly depends on channel-mediated influx of extracellular calcium.

### 3.3 Paradoxical effects of pharmacological inhibition with HC-067047 and GsMTx4 on hypotonic shock-induced calcium responses in primary astrocytes

Next, we asked whether mechanosensitive channels previously implicated in astrocyte calcium signaling contribute to hypotonic shock-induced calcium responses. Astrocyte cell swelling leads to changes in membrane tension and curvature, which can activate mechanosensitive ion channels (Cox et al., 2019). The mechanosensitive cation channels TRPV4 and Piezo1 have been implicated in astrocyte calcium signaling (Benfenati et al., 2007; Velasco-Estevez et al., 2020; Chi et al., 2022; Gomez-Cruz et al., 2024). To test their contribution pharmacologically, we treated primary astrocytes with HC-067047, a selective TRPV4 antagonist (Everaerts et al., 2010), and GsMTx4, a peptide inhibitor of cation-permeable mechanosensitive channels including Piezo channels (Bae et al., 2011). Based on the known properties of TRPV4 and Piezo1, we expected these pharmacological interventions to reduce, or at least not enhance, hypotonic shock-induced calcium responses.

**Figure 4:**
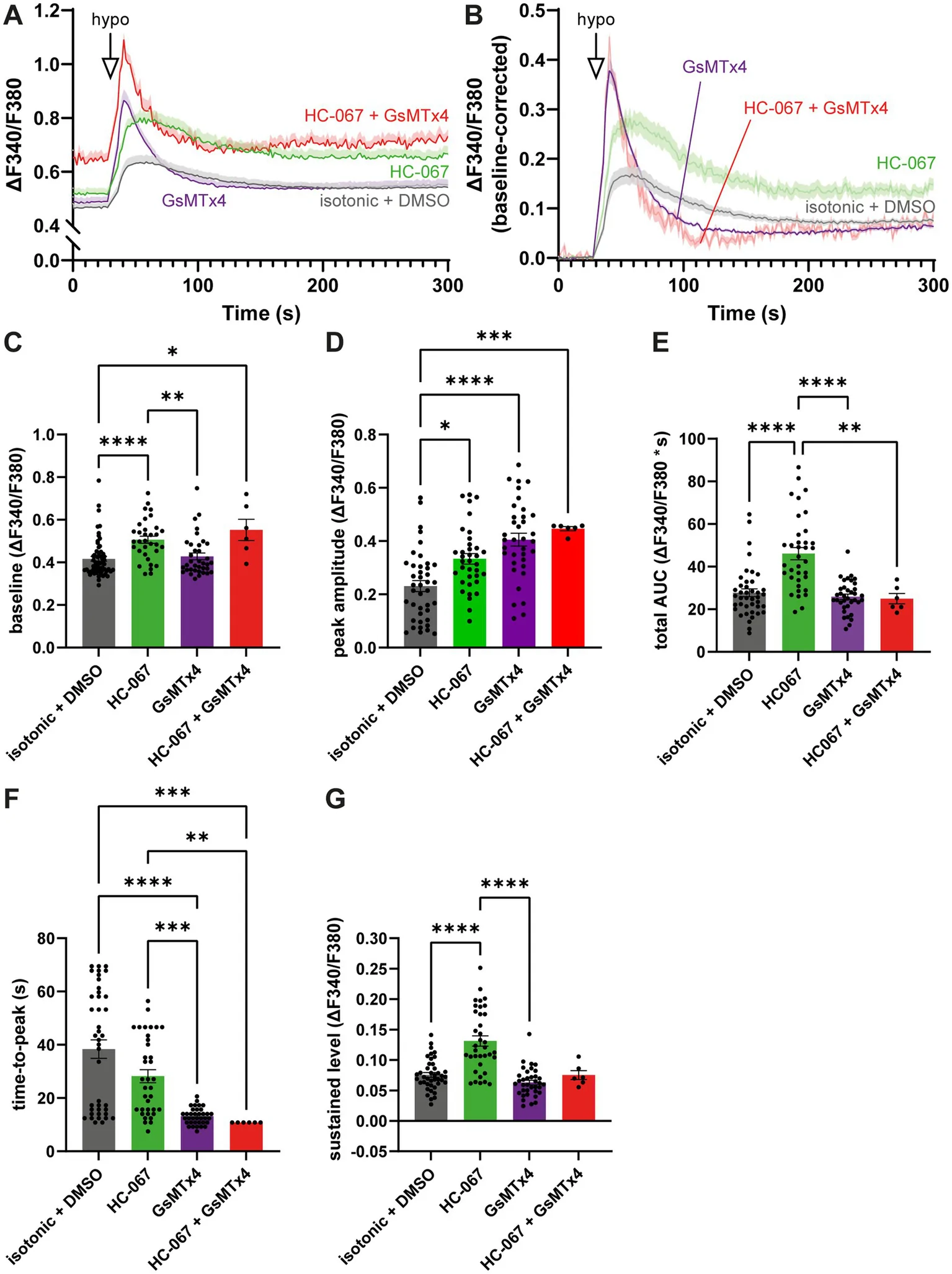
Paradoxical effects of pharmacological TRPV4 and Piezo1 inhibition on swelling-induced calcium signals in wild-type astrocytes. **(A, B)** Averaged intracellular calcium traces in wild-type astrocytes exposed to a 50% hypotonic shock (black arrow). Astrocytes were pretreated with and during imaging kept in the TRPV4 antagonist HC-067047 (green), Piezo1 inhibitor GsMTx4 (purple) or a combination of both (red). Traces in **(B)** are baseline-corrected. **(C)** Baseline calcium levels of wild-type astrocytes treated with the TRPV4 antagonist HC-067047, the Piezo1 inhibitor GsMTx4 or a combination of both (isotonic + DMSO: 0.4167 ± 0.01163 ΔF340/F380, *n* = 60 wells, HC-067047: 0.5059 ± 0.01673 ΔF340/F380, *n* = 33 wells, GsMTx4: 0.4278 ± 0.01560 ΔF340/F380, *n* = 36 wells, HC-067047 + GsMTx4: 0.5521 ± 0.05006 ΔF340/F380, *n* = 6 wells; isotonic + DMSO vs. HC-067047, adj. *p* < 0.0001, isotonic + DMSO vs. GsMTx4, adj. *p* > 0.9999, isotonic + DMSO vs. HC-067047 + GsMTx4, adj. *p* = 0.0221, HC-067047 vs. GsMTx4, adj. *p* = 0.0039, HC-067047 vs. HC-067047 + GsMTx4, adj. *p* > 0.9999, GsMTx4 vs. HC-067047 + GsMTx4, adj. *p* = 0.0697). **(D–G)** Different parameters of the calcium response to a 50% hypotonic shock in wild-type astrocytes treated with the TRPV4 antagonist HC-067047, the Piezo1 inhibitor GsMTx4 or a combination of both (isotonic + DMSO: *n* = 41 wells, HC-067047: *n* = 36 wells, GsMTx4: *n* = 36 wells, HC-067047 + GsMTx4: *n* = 6 wells). **(D)** The peak amplitude (isotonic + DMSO: 0.2311 ± 0.02045 ΔF340/F380, HC-067047: 0.3333 ± 0.01968 ΔF340/F380, GsMTx4: 0.4052 ± 0.02417 ΔF340/F380, HC-067047 + GsMTx4: 0.4466 ± 0.007613 ΔF340/F380; isotonic + DMSO vs. HC-067047, adj. *p* = 0.0145, isotonic + DMSO vs. GsMTx4, adj. *p* < 0.0001, isotonic + DMSO vs. HC-067047 + GsMTx4, adj. *p* = 0.0009, HC-067047 vs. GsMTx4, adj. *p* = 0.2015, HC-067047 vs. HC-067047 + GsMTx4, adj. *p* = 0.1754, GsMTx4 vs. HC-067047 + GsMTx4, adj. *p* > 0.9999). **(E)** The total AUC (isotonic + DMSO: 27.68 ± 1.831 ΔF340/F380 * s, HC-067047: 46.10 ± 2.88 ΔF340/F380 * s, GsMTx4: 25.87 ± 1.234 ΔF340/F380 * s, HC-067047 + GsMTx4: 24.95 ± 2.448 ΔF340/F380 * s; isotonic + DMSO vs. HC-067047, adj. *p* < 0.0001, isotonic + DMSO vs. GsMTx4, adj. *p* > 0.9999, isotonic + DMSO vs. HC-067047 + GsMTx4, adj. *p* > 0.9999, HC-067047 vs. GsMTx4, adj. *p* < 0.0001, HC-067047 vs. HC-067047 + GsMTx4, adj. *p* = 0.0091, GsMTx4 vs. HC-067047 + GsMTx4, adj. *p* > 0.9999). **(F)** The time-to-peak (TTP) (isotonic + DMSO: 38.38 ± 3.484 s, HC-067047: 28.21 ± 2.412 s, GsMTx4: 13.14 ± 0.5116 s, HC-067047 + GsMTx4: 10.8 ± 0.0 s; isotonic + DMSO vs. HC-067047, adj. *p* > 0.9999, isotonic + DMSO vs. GsMTx4, adj. *p* < 0.0001, isotonic + DMSO vs. HC-067047 + GsMTx4, adj. *p* = 0.0001, HC-067047 vs. GsMTx4, adj. *p* = 0.0001, HC-067047 vs. HC-067047 + GsMTx4, adj. *p* = 0.0019, GsMTx4 vs. HC-067047 + GsMTx4, adj. *p* > 0.9999). **(G)** The sustained calcium level (isotonic + DMSO: 0.07542 ± 0.003951 ΔF340/F380, HC-067047: 0.1313 ± 0.008535 ΔF340/F380, GsMTx4: 0.06306 ± 0.003887 ΔF340/F380, HC-067047 + GsMTx4: 0.07525 ± 0.007317 ΔF340/F380; isotonic + DMSO vs. HC-067047, adj. *p* < 0.0001, isotonic + DMSO vs. GsMTx4, adj. *p* = 0.3145, isotonic + DMSO vs. HC-067047 + GsMTx4, adj. *p* > 0.9999, HC-067047 vs. GsMTx4, adj. *p* < 0.0001, HC-067047 vs. HC-067047 + GsMTx4, adj. *p* = 0.1104, GsMTx4 vs. HC-067047 + GsMTx4, adj. *p* > 0.9999). All data is presented as mean ± SEM. Statistical analyses in **(C–G)** were done using a Kruskal-Wallis test with Dunn’s multiple comparison correction. Dots in bar graphs indicate individual values of wells, and only statistically significant comparisons are indicated in bar graphs. Individual intracellular traces are shown in Supplementary Figure 5.

In contrast, treatment with HC-067047 and/or GsMTx4 did not reduce the calcium response but instead led to clear increases in distinct aspects of swelling-induced calcium signaling (Figure 4A, baseline-corrected curves in Figure 4B). Primary astrocytes treated with the TRPV4 antagonist HC-067047 showed increased baseline calcium levels (Figure 4C). Following hypotonic shock, the total calcium response increased, reflected by an increase in peak amplitude (Figure 4D), total AUC (Figure 4E) and sustained calcium level (Figure 4G). The TTP was not affected (Figure 4F). Treatment with the mechanosensitive channel inhibitor GsMTx4 did not affect baseline calcium levels (Figure 4C), total AUC (Figure 4E), or sustained calcium level (Figure 4G). However, it increased the peak amplitude (Figure 4D) and led to a faster calcium response (Figure 4F). Combined treatment with HC-067047 and GsMTx4 increased baseline calcium levels (Figure 4C), and peak amplitude (Figure 4D). The TTP was reduced (Figure 4F), but total AUC and sustained level were not affected (Figures 4E,G).

Taken together, TRPV4 and Piezo1 are unlikely to be the main direct calcium entry pathways underlying the swelling-induced calcium response under these experimental conditions. Instead, pharmacological TRPV4 antagonism and GsMTx4-sensitive mechanosensitive channel inhibition paradoxically enhance distinct aspects of swelling-induced calcium signaling in primary astrocytes.

### 3.4 Basal calcium and hypotonic-induced calcium dynamics in *Mlc1*-null astrocytes are altered

Astrocyte volume regulation is affected in the leukodystrophy MLC, as primary astrocytes from *Mlc1*-null mice have a disturbed RVD (Ridder et al., 2011; Dubey et al., 2015). Intracellular calcium is an important trigger for the RVD process in several cell types (Pasantes-Morales and Morales Mulia, 2000), and MLC1 was recently suggested to interact with intracellular calcium (Brignone et al., 2024). Therefore, we investigated baseline and swelling-induced calcium dynamics in *Mlc1*-null astrocytes (Figure 5A, baseline-corrected curves in Figure 5B). Baseline calcium levels were increased in *Mlc1*-null astrocytes (Figure 5C). There was no difference in the response to a 30% hypotonic shock between wild-type and *Mlc1*-null astrocytes (Figures 5D–G). However, the calcium response to a 50% hypotonic shock was significantly slower in *Mlc1*-null astrocytes (Figure 5J), and the sustained calcium level was slightly reduced in these cells (Figure 5K) when compared to wild-type astrocytes. Peak amplitudes and total AUC were not altered (Figures 5H,I). Together, these data indicate that loss of MLC1 leads to increased baseline astrocyte calcium levels and affects the speed of the calcium response to strong hypotonic stimulation.

### 3.5 *Mlc1*-null astrocytes show differences in TRPV4-mediated calcium dynamics

Finally, we asked whether loss of MLC1 alters the effects of pharmacological TRPV4 antagonism or GsMTx4-sensitive mechanosensitive channel inhibition on hypotonicity-induced calcium signaling. To address whether genotype-dependent differences could be related to altered expression of the pharmacologically targeted channels, we first considered available expression data. In our previously published proteomic analysis, total Piezo1 protein abundance was not significantly different between wild-type and *Mlc1*-null astrocytes (Bisseling et al., 2026). In addition, western blot analysis did not show a significant difference in total TRPV4 protein expression between wild-type and *Mlc1*-null astrocytes, although a small trend toward higher TRPV4 expression was observed in *Mlc1*-null astrocytes (Supplementary Figure 2). These data argue against major genotype-dependent changes in total Piezo1 or TRPV4 protein abundance, although they do not exclude differences in channel (membrane) localization or functional coupling.

**Figure 6:**
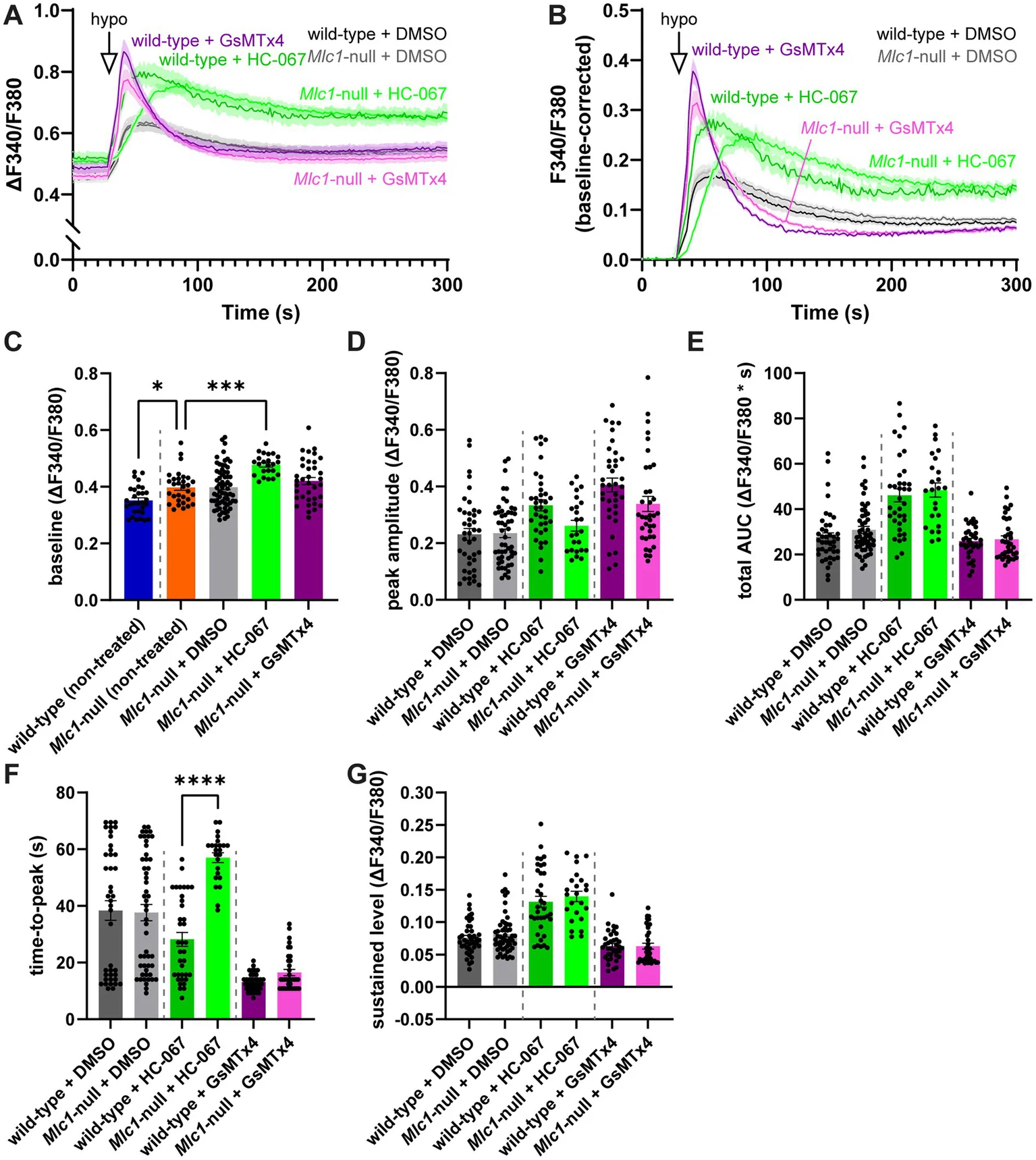
The effect of pharmacological inhibition of TRPV4 and Piezo1 on swelling-induced calcium signals in wild-type versus *Mlc1*-null astrocytes. **(A, B)** Averaged intracellular calcium traces in wild-type (green, purple) and *Mlc1*-null (light green, pink) astrocytes exposed to a 50% hypotonic shock (black arrow). Astrocytes were pretreated with and during imaging kept in TRPV4 antagonist HC-067047 (green) or Piezo1 inhibitor GsMTx4 (purple). Traces in **(B)** are baseline-corrected. **(C)** Baseline calcium levels of wild-type and *Mlc1*-null astrocytes treated with the TRPV4 antagonist HC-067047 or the Piezo1 inhibitor GsMTx4 (wild-type: 0.3519 ± 0.009295 ΔF340/F380, *n* = 30 wells, *Mlc1*-null: 0.3977 ± 0.01022 ΔF340/F380, *n* = 31 wells, *Mlc1*-null + DMSO: 0.3983 ± 0.008810 ΔF340/F380, *n* = 66 wells, *Mlc1*-null + HC-067047: 0.4775 ± 0.007601 ΔF340/F380, *n* = 23 wells, *Mlc1*-null + GsMTx4: 0.4206 ± 0.01261 ΔF340/F380, *n* = 36 wells; wild-type vs. *Mlc1*-null, adj. *p* = 0.0486, *Mlc1*-null vs. *Mlc1*-null + DMSO, adj. *p* > 0.9999, *Mlc1*-null vs. *Mlc1*-null + HC-067047, adj. *p* = 0.0001, *Mlc1*-null vs. *Mlc1*-null + GsMTx4, adj. *p* = 0.9765). **(D–G)** Different parameters of the calcium response to a 50% hypotonic shock in wild-type and *Mlc1*-null astrocytes treated with the TRPV4 antagonist HC-067047 or the Piezo1 inhibitor GsMTx4 (wild-type + DMSO: *n* = 41 wells, *Mlc1*-null + DMSO: *n* = 51 wells, wild-type + HC-067047: *n* = 36 wells, *Mlc1*-null + HC-067047: *n* = 24 wells, wild-type + GsMTx4: *n* = 36 wells, *Mlc1*-null + GsMTx4: *n* = 36 wells). **(D)** The peak amplitude (wild-type + DMSO: 0.2311 ± 0.02045 ΔF340/F380, *Mlc1*-null + DMSO: 0.2347 ± 0.01505 ΔF340/F380, adj. *p* > 0.9999, wild-type + HC-067047: 0.3333 ± 0.01968 ΔF340/F380, *Mlc1*-null + HC-067047: 0.2613 ± 0.01920 ΔF340/F380, adj. *p* = 0.1597, wild-type + GsMTx4: 0.4052 ± 0.02417 ΔF340/F380, *Mlc1*-null + GsMTx4: 0.3384 ± 0.02642 ΔF340/F380, adj. *p* = 0.0637). **(E)** The total AUC (wild-type + DMSO: 27.68 ± 1.831 ΔF340/F380 * s, *Mlc1*-null + DMSO: 30.84 ± 1.539 ΔF340/F380 * s, adj. *p* = 0.4763, wild-type + HC-067047: 46.10 ± 2.880 ΔF340/F380 * s, *Mlc1*-null + HC-067047: 48.40 ± 3.137 ΔF340/F380 * s, adj. *p* > 0.9999, wild-type + GsMTx4: 25.87 ± 1.234 ΔF340/F380 * s, *Mlc1*-null + GsMTx4: 26.68 ± 1.523 ΔF340/F380 * s, adj. *p* > 0.9999). **(F)** The time-to-peak (TTP) (wild-type + DMSO: 38.38 ± 3.484 s, *Mlc1*-null + DMSO: 37.66 ± 2.865 s, adj. *p* > 0.9999, wild-type + HC-067047: 28.21 ± 2.412 s, *Mlc1*-null + HC-067047: 57.03 ± 1.707 s, adj. *p* < 0.0001, wild-type + GsMTx4: 13.14 ± 0.5116 s, *Mlc1*-null + GsMTx4: 16.48 ± 1.095 s, adj. *p* = 0.6913). **(G)** The sustained calcium level (wild-type + DMSO: 0.07542 ± 0.003951 ΔF340/F380, *Mlc1*-null + DMSO: 0.08138 ± 0.004231 ΔF340/F380, adj. *p* > 0.9999, wild-type + HC-067047: 0.1313 ± 0.008535 ΔF340/F380, *Mlc1*-null + HC-067047: 0.1396 ± 0.008069 ΔF340/F380, adj. *p* > 0.9999, wild-type + GsMTx4: 0.06306 ± 0.003887 ΔF340/F380, *Mlc1*-null + GsMTx4: 0.06280 ± 0.004486 ΔF340/F380, adj. *p* > 0.9999). All data is presented as mean ± SEM. Statistical analyses in **(C–G)** were done using a Kruskal–Wallis test with Dunn’s multiple comparison correction. Dots in bar graphs indicate individual values of wells, and only statistically significant comparisons are indicated in bar graphs. Individual intracellular traces of *Mlc1*-null astrocytes are shown in Supplementary Figure 6.

We then treated *Mlc1*-null primary astrocytes with the TRPV4 antagonist HC-067047 or the mechanosensitive channel inhibitor GsMTx4 (Figure 6A, baseline-corrected curves in Figure 6B), to investigate how hypotonicity-induced calcium signals were affected. Baseline calcium levels were significantly increased by TRPV4 antagonism, compared to the already increased baseline of untreated *Mlc1*-null astrocytes (Figure 6C). This is similar to the increase in baseline calcium that was observed in wild-type astrocytes (Figure 4C). Baseline calcium was not affected by GsMTx4 again similar to what was observed in wild-type astrocytes. Surprisingly, TRPV4 antagonism in *Mlc1*-null astrocytes slowed down the calcium response to a hypotonic shock, nearly doubling the TTP (Figure 6F). In contrast, no such effect was observed in wild-type astrocytes (Figures 4F and 6F). The same trend was observed when giving a 30% hypotonic shock (data not shown).

Other properties of the calcium signal after HC-067047 or GsMTx4 treatment were not different between wild-type and *Mlc1*-null astrocytes (Figures 6D,E,G). GsMTx4 treatment showed a trend toward a smaller increase in peak amplitude in *Mlc1*-null astrocytes compared to wild-type, but this difference did not reach statistical significance (Figure 6D). Together, these results indicate that loss of MLC1 alters the response to pharmacological TRPV4 antagonism, particularly by further slowing the already delayed calcium response to hypotonic shock. In contrast, responses to GsMTx4-sensitive mechanosensitive channel inhibition were not clearly different between wild-type and *Mlc1*-null astrocytes.

## 4 Discussion

How calcium signaling contributes to astrocyte volume regulation remains poorly understood. Here, we investigated how calcium from various sources, such as calcium influx through calcium channels and calcium release from stores, contributes to calcium signaling in response to osmotic changes in astrocytes. We show that cultured wild-type primary astrocytes generate robust calcium responses to hypotonic swelling. As expected, these responses scale with the intensity of the shock and involve both calcium release from intracellular stores and extracellular calcium influx. Unexpectedly, pharmacological treatment with the TRPV4 antagonist HC-067047 and the mechanosensitive channel inhibitor GsMTx4 did not reduce swelling-induced calcium signals but enhanced distinct aspects of the response. These findings suggest that TRPV4- and GsMTx4-sensitive pathways modulate astrocyte responsiveness to osmotic stress, while arguing against TRPV4 or Piezo1 being the main direct calcium entry routes under these experimental conditions. Compared to wild-type astrocytes, *Mlc1*-null astrocytes showed elevated basal calcium levels, slower calcium responses to strong hypotonic stimulation, and altered responses to TRPV4 antagonism.

As shown before (O'Connor and Kimelberg, 1993; Fischer et al., 1997; Morales-Mulia et al., 1998; Benfenati et al., 2007), hypotonic shock-induced astrocyte swelling induced a calcium response with an initial transient increase, followed by a sustained phase. Our experiments indicate that this response depends on both intracellular calcium stores and extracellular calcium influx. Removal of extracellular calcium mainly reduced the sustained phase, while depletion of ER calcium stores with thapsigargin affected the initial peak. However, thapsigargin treatment in the presence of extracellular calcium is difficult to interpret in isolation, because ER calcium depletion can trigger SOCE (Verkhratsky and Parpura, 2014). Therefore, the combined thapsigargin and zero calcium condition provides the clearest evidence for a contribution of ER calcium stores. Only the combination of both manipulations almost completely abolished the calcium signal, supporting the conclusion that the initial phase depends on intracellular store release and that the sustained phase largely depends on extracellular calcium influx. Inhibition of PLC with U-73122 did not measurably alter the response, suggesting that PLC-dependent IP_3_-mediated store release is not a major pathway involved here. However, because this conclusion is based on pharmacological inhibition, and because U-73122 has known limitations, this negative result should be interpreted cautiously.

The paradoxical effects of HC-067047 and GsMTx4 argue against a simple model in which TRPV4 and Piezo1 are the main calcium entry routes during hypotonic stimulation in our assay. If these channels were the dominant source of calcium influx, their pharmacological inhibition would be expected to reduce the response. Instead, both compounds enhanced distinct aspects of swelling-induced calcium signaling. This suggests that the targeted pathways may influence how astrocytes are primed to respond to osmotic stress, rather than directly carrying the swelling-induced calcium influx.

For TRPV4, the interpretation is relatively direct because HC-067047 is widely used as a selective channel antagonist. TRPV4 has been linked to calcium influx in response to hypotonicity-induced astrocyte swelling in several studies (Benfenati et al., 2007; Benfenati et al., 2011; White et al., 2016). However, HC-067047 did not suppress the swelling-induced calcium response in our experiments, but instead increased baseline calcium levels and enhanced the peak amplitude and sustained component of the response. This suggests that basal TRPV4 activity may help maintain a cellular state that limits astrocyte calcium activity. In line with this interpretation, Eilert-Olsen et al. reported that TRPV4 inhibition increased spontaneous calcium activity in astrocyte endfeet under normosmotic conditions in intact tissue, although they did not observe an enhanced response to hypoosmotic stimulation (Eilert-Olsen et al., 2019). The relationship between TRPV4 activity, basal calcium signaling and swelling-induced calcium responses is therefore likely context dependent. A possible mechanistic explanation is that basal TRPV4 activity contributes to the tuning of membrane mechanics or cytoskeletal organization. TRPV4 is tethered to the cytoskeleton (Ramadass et al., 2007; Goswami et al., 2010), and forms a complex with cytoskeletal regulator RhoA, which is dissociated upon hypotonicity or TRPV4 activation (McCray et al., 2021). Acute TRPV4 antagonism with HC-067047 could therefore shift the mechanical or cytoskeletal state of astrocytes before the hypotonic challenge, thereby lowering the threshold for a larger calcium response to swelling.

The interpretation of the GsMTx4 experiments requires more caution. Targeting Piezo1 was the main rationale for using GsMTx4 in these experiments, because Piezo1 is directly gated by membrane tension (Coste et al., 2010; Cox et al., 2016) and has been implicated in astrocyte mechanotransduction (Velasco-Estevez et al., 2020; Liu et al., 2021; Chi et al., 2022; Gomez-Cruz et al., 2024). However, the paradoxical increase in calcium responses after GsMTx4 treatment does not support a simple model in which Piezo1 acts as the main calcium entry route during hypotonic stimulation. Moreover, GsMTx4 is not a selective Piezo1 antagonist. Rather, it is a peptide modulator of cation-permeable mechanosensitive channels, including Piezo channels, and its effects are thought to involve interactions with the lipid membrane and altered force transmission to mechanosensitive channels rather than classical receptor antagonism (Bae et al., 2011; Gnanasambandam et al., 2017). In addition, both inhibitory and potentiating effects of GsMTx4 on mechanosensitive channels have been reported (Suchyna, 2017). Our data should therefore be interpreted as evidence that a GsMTx4-sensitive mechanism modulates swelling-induced calcium signaling, rather than as definitive evidence for a Piezo1-specific effect. In contrast to HC-067047, GsMTx4 did not alter baseline calcium levels, but increased peak amplitude twofold and accelerated the response to hypotonic shock. This suggests that the GsMTx4-sensitive effect is mechanistically distinct from the HC-067047-sensitive effect. The present pharmacological experiments cannot distinguish whether this reflects modulation of Piezo1, another mechanosensitive channel, or a more general membrane-mediated effect of GsMTx4.

*Mlc1*-null astrocytes showed elevated baseline calcium levels and a slower response to strong hypotonic stimulation. The overall amplitude of the response was largely preserved, indicating that loss of MLC1 does not abolish swelling-induced calcium signaling, but alters its basal set point and temporal organization. This is in line with previous work showing disturbed volume regulation in MLC models and altered calcium dynamics in relation to MLC1 (Ridder et al., 2011; Lanciotti et al., 2012; Dubey et al., 2015; Brignone et al., 2022; Passchier et al., 2023; Brignone et al., 2024). It also aligns with our recent finding that *Mlc1*-null astrocytes have altered mechanical properties and are softer (Bisseling et al., 2026). These structural differences could contribute to the altered calcium signaling that we find here and could further explain changes in calcium response kinetics without impacting the amplitude of the response.

The altered effects of TRPV4 antagonism in *Mlc1*-null astrocytes further support the idea that MLC1 influences the cellular context in which mechanosensitive signaling occurs. Treatment with HC-067047 substantially slowed the already delayed response of *Mlc1*-null astrocytes, an effect not observed in wild-type cells. This effect is not due to changes in TRPV4 protein expression, as there were no significant differences found between wild-type and *Mlc1*-null astrocytes. Previous studies have suggested functional interactions between MLC1, TRPV4, VRAC and calcium-dependent signaling pathways (Lanciotti et al., 2012; Brignone et al., 2014; Brignone et al., 2022; Brignone et al., 2024). Our data do not establish a direct molecular interaction between MLC1 and TRPV4 but suggest that loss of MLC1 changes how astrocytes respond to perturbation of mechanosensitive ion channels.

This study has several limitations. First, calcium signals were measured in a plate reader, providing robust population-level measurements but no information on single-cell heterogeneity or subcellular calcium microdomains. Although this assay required fluid injection and mixing, the primary astrocytes were measured as adherent cultures. The injection speed was kept low to minimize mechanical disturbance and prevent detachment during hypotonic stimulation. Second, the cultured primary astrocytes used in this study do not recapitulate the complex *in vivo* morphology of astrocytes. This is particularly relevant, as calcium dynamics can differ between soma, fine processes and perivascular endfeet (Eilert-Olsen et al., 2019), with the latter being the primary site where MLC1, AQP4, TRPV4 and other volume-regulatory proteins are enriched (Boor et al., 2005; Benfenati et al., 2007; Dubey et al., 2015). In addition, astrocytes *in vivo* are embedded in a softer and more complex extracellular environment than cells cultured on coated plastic, and they interact with blood vessels, neurons and other glial cells. These differences may affect membrane tension, cytoskeletal organization and force transduction, all of which are relevant for mechanosensitive channel activity. Therefore, the pharmacological effects observed here may not translate directly to intact tissue, where swelling-induced calcium signals may be more spatially restricted, for example to local microdomains in astrocyte processes or endfeet. Third, we did not directly measure cell volume changes or RVD alongside calcium signals and therefore cannot determine to what extent the observed calcium dynamics causally contribute to volume recovery. Finally, the role of TRPV4- and Piezo1-related pathways was assessed using pharmacological approaches. While HC-067047 is widely used as a selective TRPV4 antagonist and GsMTx4 as an inhibitor/modulator of cation-permeable mechanosensitive channels (Everaerts et al., 2010; Gnanasambandam et al., 2017), such approaches cannot distinguish direct effects on calcium entry from indirect changes in cellular properties, such as membrane tension, cytoskeletal organization or channel coupling. Future studies combining genetic perturbation (e.g., siRNA-mediated channel knockdown) with high-resolution calcium imaging in intact brain slices will be needed to further resolve how mechanosensitive channels modulate astrocyte responsiveness to osmotic stress.

**Figure 7:**
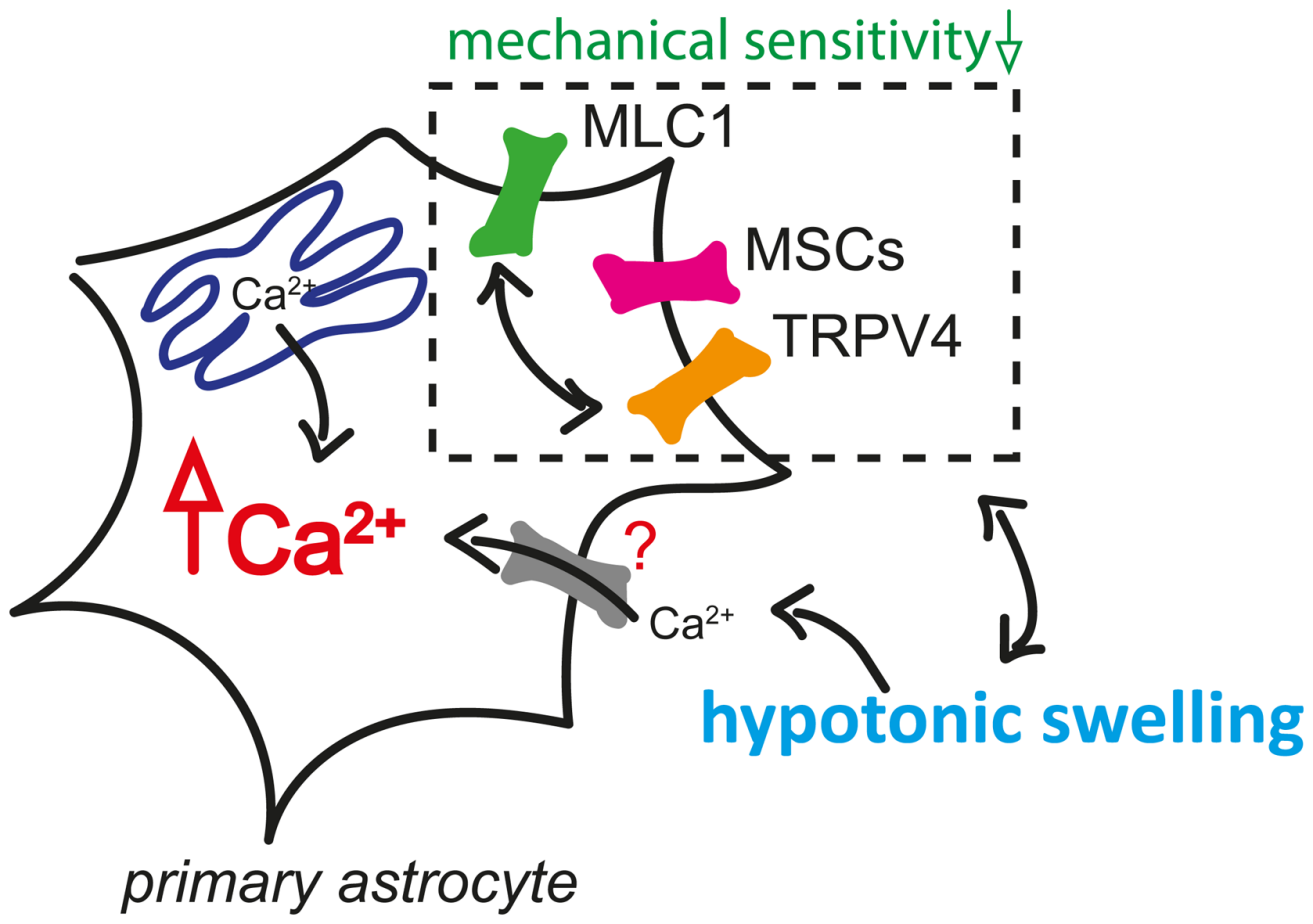
TRPV4- and GsMTx4-sensitive mechanosensitive channels (MSCs) tune astrocyte responsiveness to osmotic stress, responding differently to hypotonic swelling stimulus in presence or absence of MLC1.

In conclusion, swelling-induced calcium signaling in primary astrocytes arises from a combination of extracellular calcium influx and release from intracellular stores. Pharmacological treatment with HC-067047 and GsMTx4 has paradoxical effects: rather than reducing these responses, both compounds enhance distinct aspects of swelling-induced calcium signaling. These findings argue against TRPV4 or Piezo1 acting as the main direct calcium entry routes under these experimental conditions, and instead suggest that TRPV4- and GsMTx4-sensitive pathways influence how astrocytes translate osmotic stress into calcium signals. Loss of MLC1 shifts baseline calcium levels and alters response kinetics, indicating that MLC1 also contributes to the cellular context that couples swelling to calcium signaling. Together, these findings suggest that MLC1, TRPV4 and GsMTx4-sensitive mechanosensitive channels contribute to defining the cellular state that determines how astrocytes respond to osmotic stress (Figure 7).

## Data Availability

The raw data supporting the conclusions of this article will be made available by the authors, without undue reservation.
