# Supplementary material for: Swelling-induced calcium signaling in wild-type and Mlc1-null astrocytes: paradoxical effects of pharmacological TRPV4 and Piezo1 inhibition
Source: Front Cell Neurosci. 2026 Aug 13;20:1908522. doi: 10.3389/fncel.2026.1908522 (PMC13518189; doi:10.3389/fncel.2026.1908522)
Supplement: Supplementary file 1 [file Data_sheet_1.pdf]

Supplementary Material

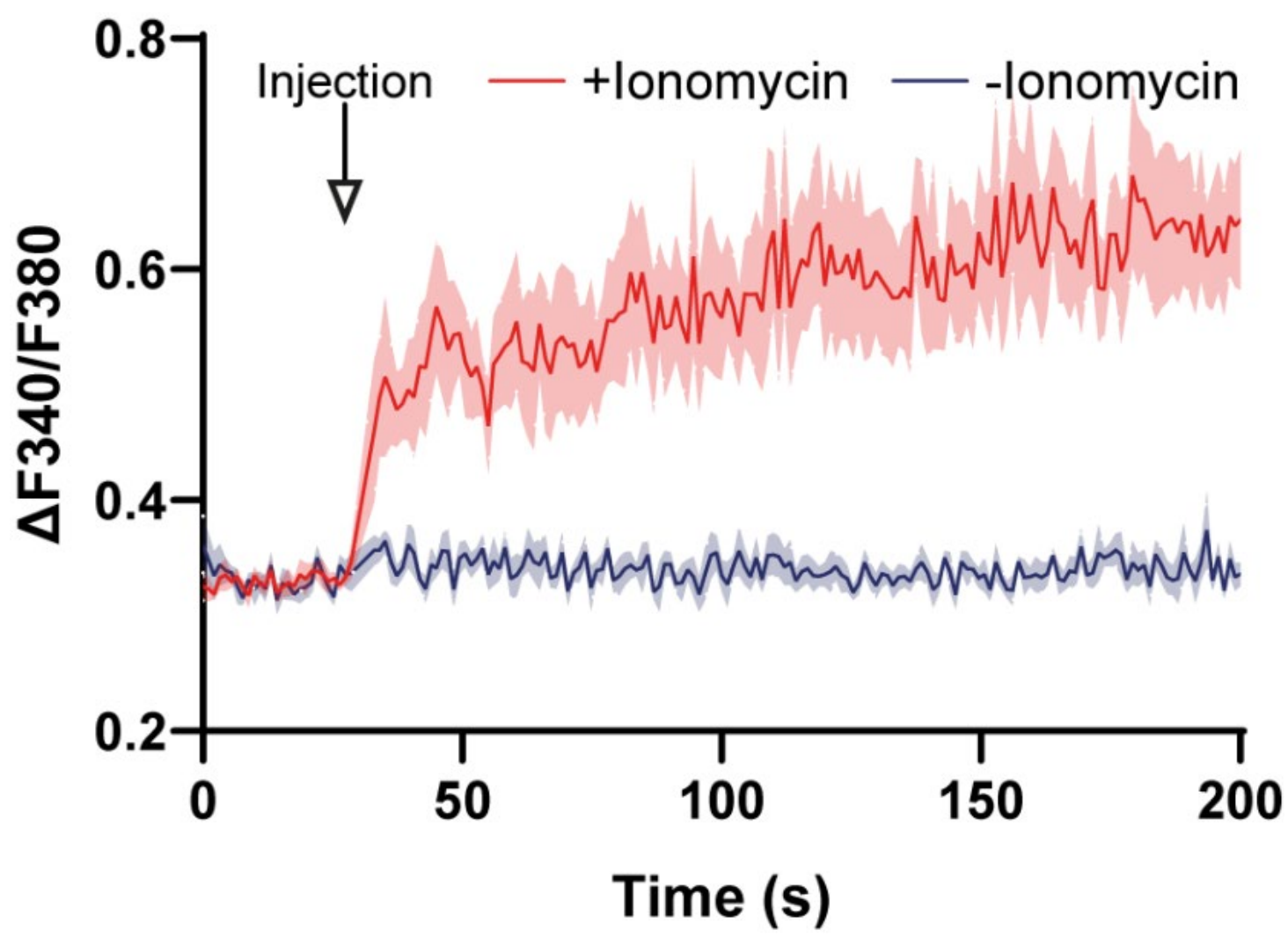

Supplementary Figure 1. Optimization of plate reader-based calcium imaging using Fura-2AM in HEK293 cells. Wells containing HEK293 cells loaded with Fura-2AM received a fluid injection of HBSS$^{-/-}$ with DMSO or HBSS$^{-/-}$ with ionomycin (0.33 mM in DMSO; arrow). Application of ionomycin induced a robust intracellular calcium increase, compared to stable baseline calcium levels in absence of ionomycin, showing that Fura-2AM is a suitable calcium detector dye (steady state calcium levels: -Ionomycin: 0.3401±0.0007853 ΔF340/F380, *n*=5 wells, +Ionomycin: 0.5911±0.003785 ΔF340/F380, *n*=5 wells, *P*<0.0001). Data is presented as mean ± SEM. Statistical analysis was done using an unpaired Student's t-test.

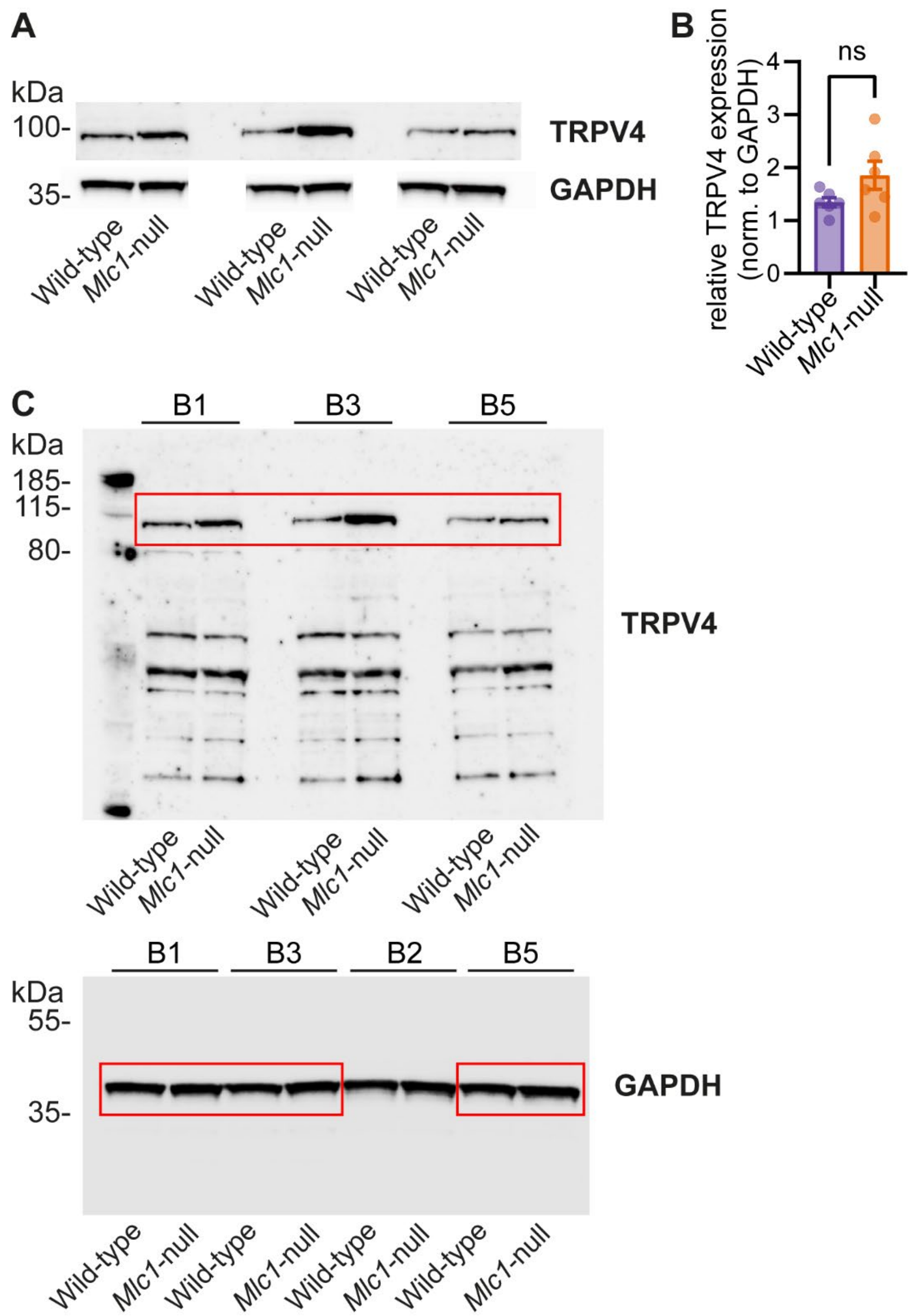

Supplementary Figure 2. Western blot analysis of TRPV4 protein expression in wild-type and *Mlc1*-null astrocytes. **(A)** Western blot of TRPV4 protein in three different preparations of wild-type and *Mlc1*-null astrocytes. **(B)** Quantification of TRPV4 western blots in wild-type (purple) and *Mlc1*-null (orange) astrocytes (Relative protein expression: Wild-type: 1.346±0.08821, *Mlc1*-null: 1.858±0.2656, *n*=6, *P*=0.0974). Bar graph shows densitometric analysis of protein expression normalized against GAPDH, which was used as a loading control, from corresponding samples. All data is presented as mean ± SEM, with dots indicating individual values in bar graphs. **(C)** Original blots of TRPV4 and GAPDH, in which red boxes outline the western blot images used in **(A)**. Molecular weight markers are indicated on the left in kilodalton (kDa).

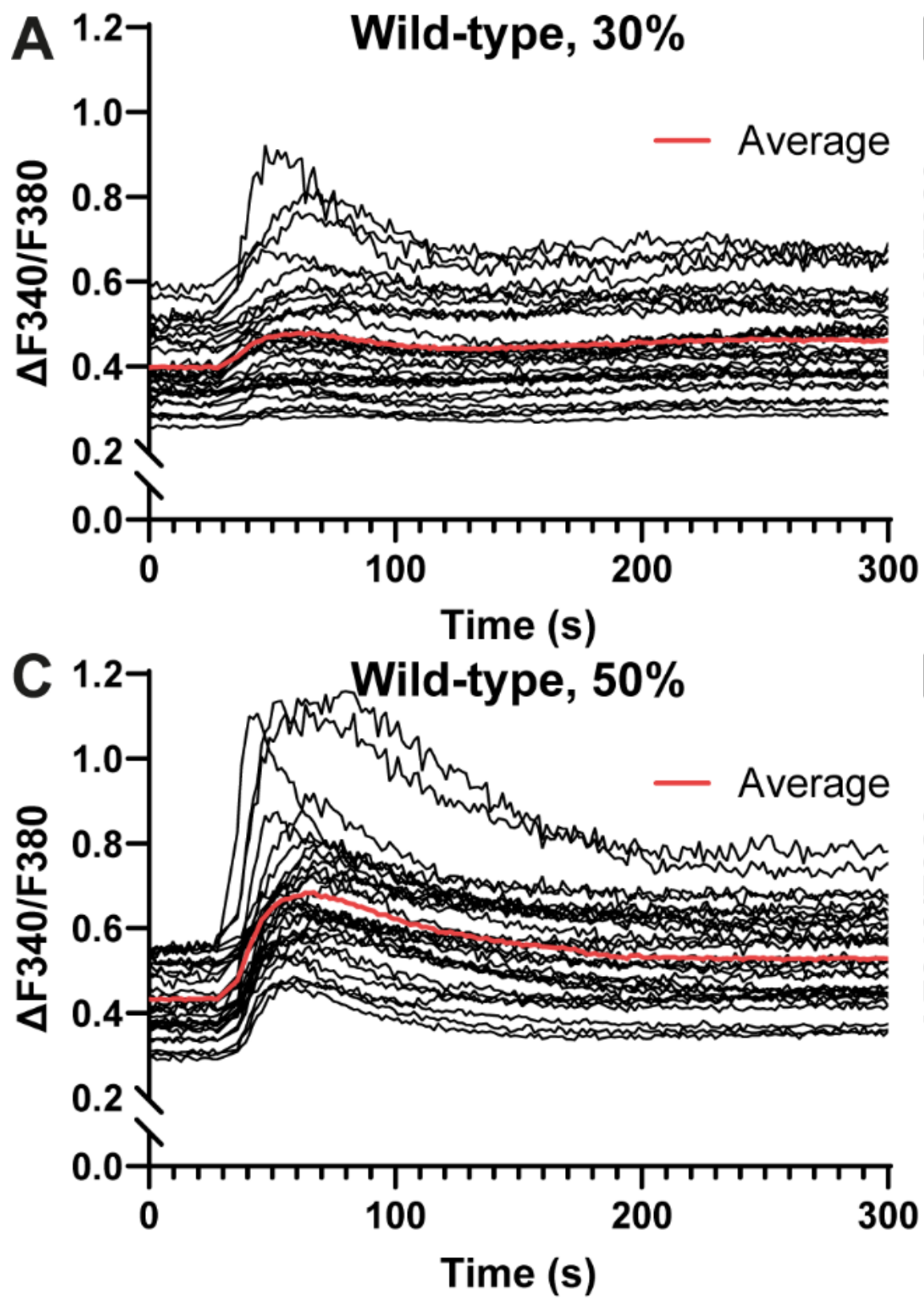

Supplementary Figure 3. Individual intracellular calcium traces from Figure 1 and 5, with corresponding averages. (A-D) Intracellular calcium traces of all individual wells (black traces) in untreated wild-type astrocytes exposed to a 30% **(A)**, 40% **(B)**, 50% **(C)** and no shock **(D)**. Each graph contains the average (red) of all individual traces.

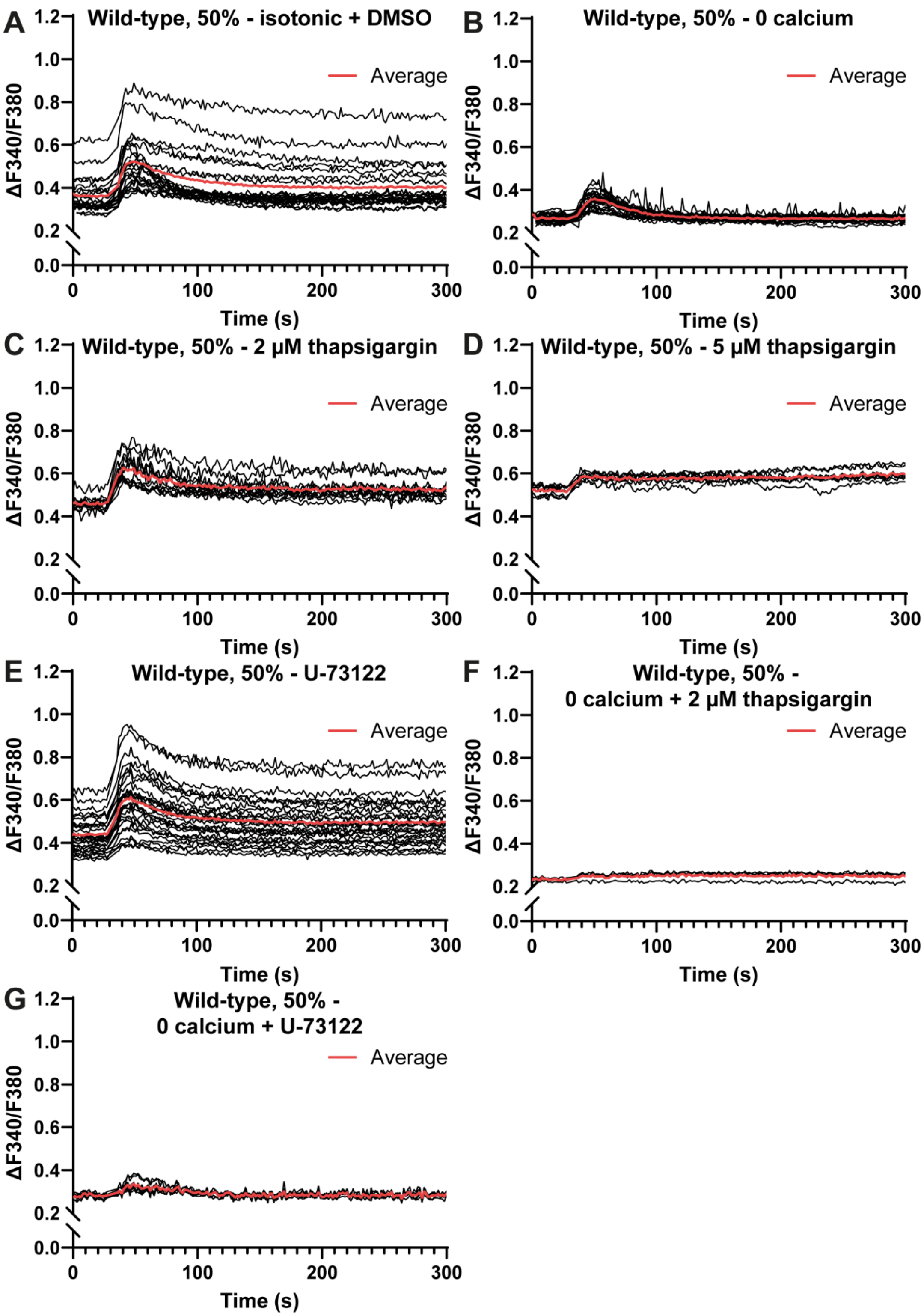

Supplementary Figure 4. Individual intracellular calcium traces from Figure 2 and 3, with corresponding averages. (A-G) Intracellular calcium traces of all individual wells (black traces) in wild-type astrocytes exposed to a 50% shock, untreated in isotonic solution with DMSO **(A)** or treated with zero calcium **(B)**, thapsigargin **(C,D)**, U-73122 **(E)**, zero calcium and thapsigargin **(F)**, and zero calcium and U-73122 **(G)**. Each graph contains the average (red) of all individual traces.

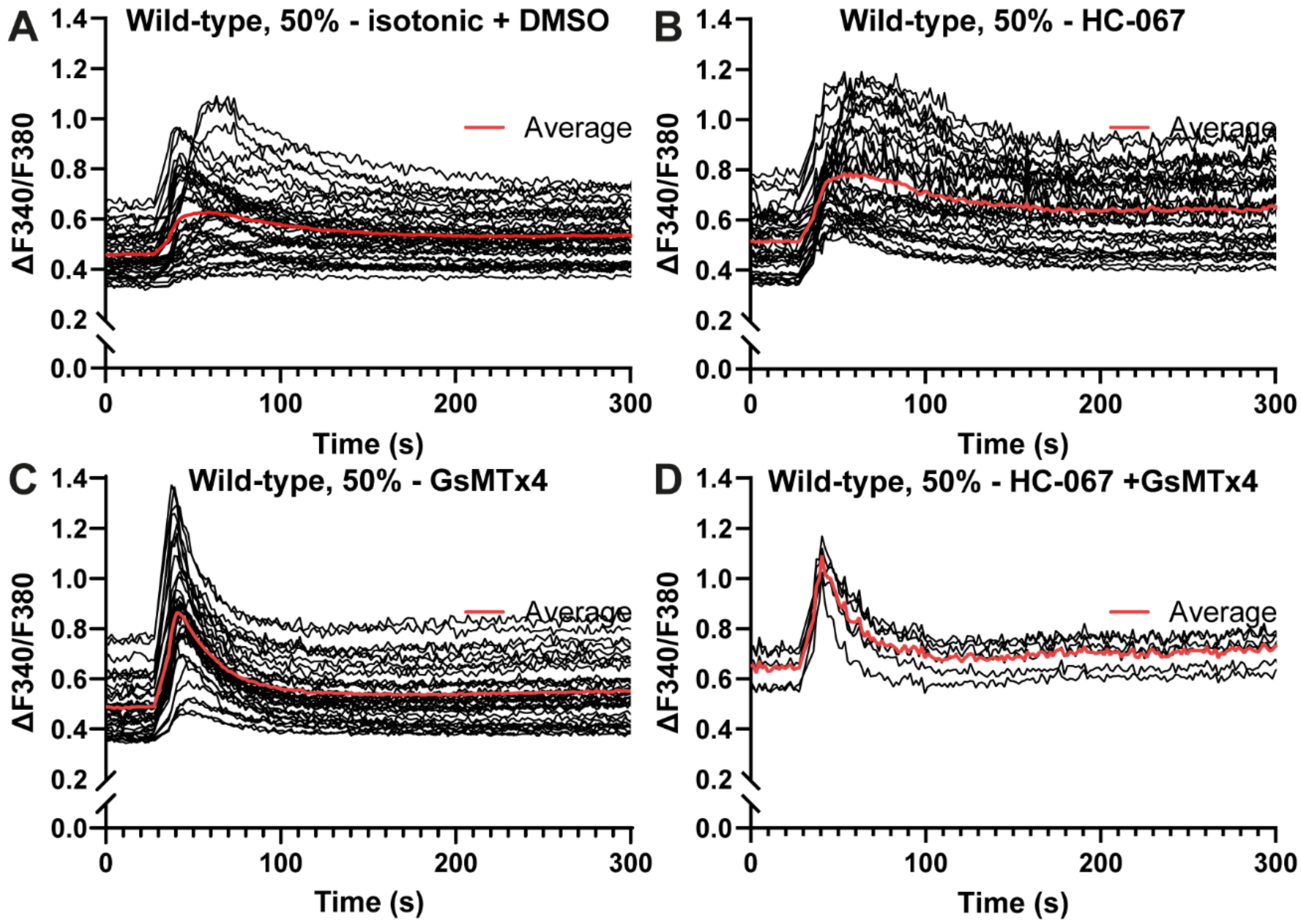

Supplementary Figure 5. Individual intracellular calcium traces from Figure 4, with corresponding averages. (A-D) Intracellular calcium traces of all individual wells (black traces) in wild-type astrocytes exposed to a 50% shock, untreated in isotonic solution with DMSO **(A)** or treated with TRPV4 antagonist HC-067047 **(B)**, inhibitor of cation permeable mechanosensitive ion channels GsMTx4 (which blocks Piezo1) **(C)**, and both HC-067047 and GsMTx4 **(D)**. Each graph contains the average (red) of all individual traces.

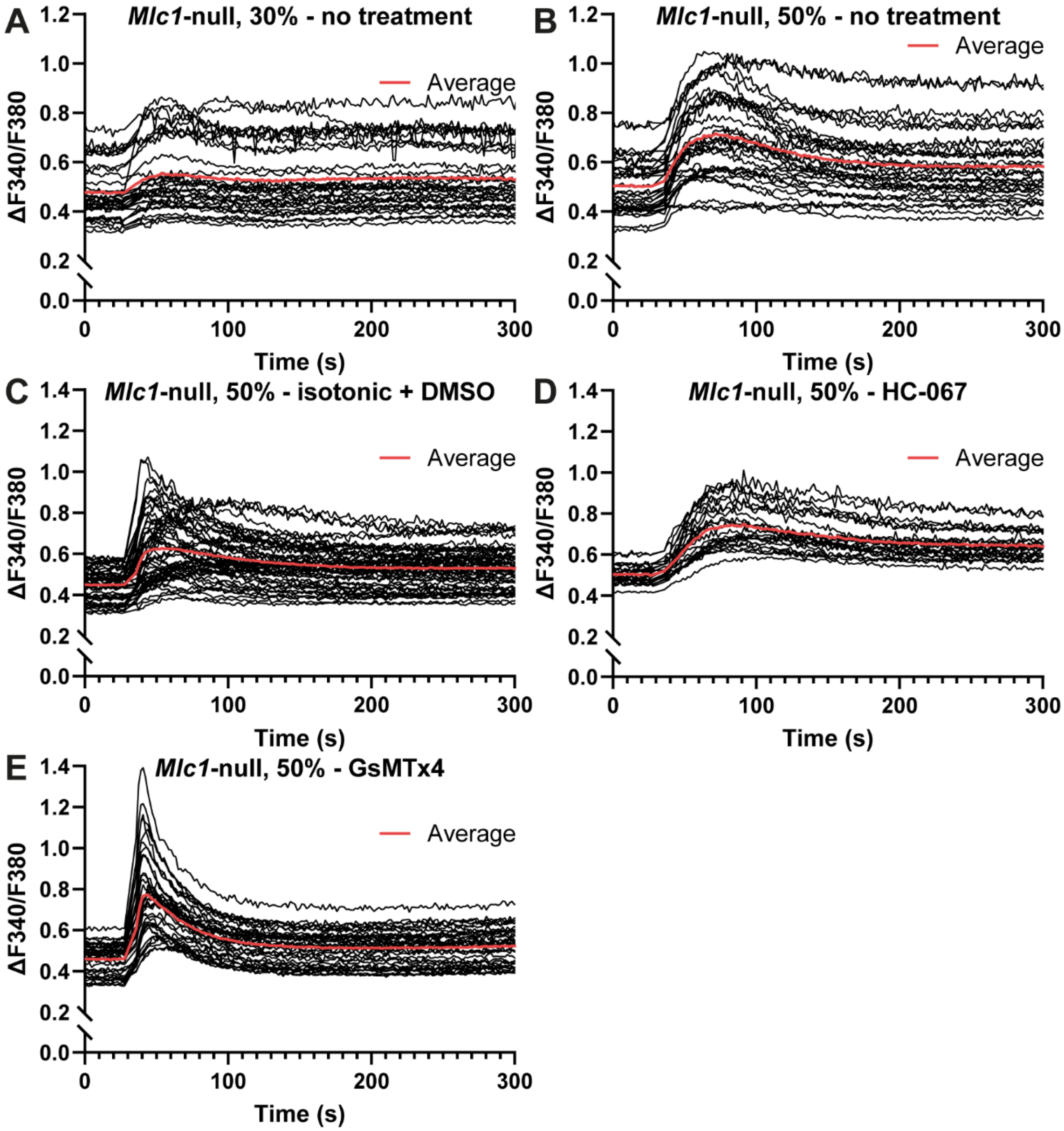

Supplementary Figure 6. Individual intracellular calcium traces of all *Mlc1*-null astrocytes from Figures 5 and 6, with corresponding averages. (A-E) Intracellular calcium traces of all individual wells (black traces) in *Mlc1*-null astrocytes. **(A,B)** Untreated in isotonic solution exposed to a 30% **(A)** or 50% **(B)** hyposhock. **(C-E)** Cells exposed to a 50% shock, untreated in isotonic solution with DMSO **(C)**, or treated with TRPV4 antagonist HC-067047 **(D)** or with inhibitor of cation permeable mechanosensitive ion channels GsMTx4 **(E)**. Each graph contains the average (red) of all individual traces.
